# A Megafauna’s Microfauna: Gastrointestinal Parasites of New Zealand’s Extinct Moa (Aves: Dinornithiformes)

**DOI:** 10.1371/journal.pone.0057315

**Published:** 2013-02-25

**Authors:** Jamie R. Wood, Janet M. Wilmshurst, Nicolas J. Rawlence, Karen I. Bonner, Trevor H. Worthy, John M. Kinsella, Alan Cooper

**Affiliations:** 1 Landcare Research, Lincoln, Canterbury, New Zealand; 2 Australian Centre for Ancient DNA, School of Earth and Environmental Sciences, University of Adelaide, Adelaide, South Australia, Australia; 3 School of Biological, Earth and Environmental Sciences, University of New South Wales, Sydney, Australia; 4 HelmWest Laboratory, Missoula, Montana, United States of America; Raymond M. Alf Museum of Paleontology, United States of America

## Abstract

We perform the first multidisciplinary study of parasites from an extinct megafaunal clade using coprolites from the New Zealand moa (Aves: Dinornithiformes). Ancient DNA and microscopic analyses of 84 coprolites deposited by four moa species (South Island giant moa, *Dinornis robustus*; little bush moa, *Anomalopteryx didiformis*; heavy-footed moa, *Pachyornis elephantopus*; and upland moa, *Megalapteryx didinus*) reveal an array of gastrointestinal parasites including coccidians (*Cryptosporidium* and members of the suborder Eimeriorina), nematodes (Heterakoidea, Trichostrongylidae, Trichinellidae) and a trematode (Echinostomida). Parasite eggs were most prevalent and diverse in coprolites from lowland sites, where multiple sympatric moa species occurred and host density was therefore probably higher. Morphological and phylogenetic evidence supports a possible vicariant Gondwanan origin for some of the moa parasites. The discovery of apparently host-specific parasite taxa suggests paleoparasitological studies of megafauna coprolites may provide useful case-studies of coextinction.

## Introduction

Coprolites (preserved faecal boli) are rich sources of paleoecological information. Historically, the main focus of coprolite studies has been dietary reconstruction [1]–[5]. However, paleoparasitological analyses have also been reported for a large number of coprolites, both archaeological and paleontological in origin [6], and from a broad range of vertebrate taxa including dinosaurs [7], rodents [8], ground sloth [9], lizards [10], carnivores [11], [12], birds [13] and humans [14]–[16]. Identification of parasites in coprolites can provide information on the ecology [17], population dynamics [18] and diseases of prehistoric animals, and the evolution of host-parasite relationships [19], [20].

During the past fifteen years, advances in ancient DNA (aDNA) techniques have provided increased potential for the application of molecular paleoparasitology to coprolites, yet implementation has so far been limited [20], [21]. The potential benefits of aDNA analysis, when used in conjunction with conventional microscopic techniques, include better taxonomic resolution (particularly if only eggs are present) and detection of very small parasites (e.g. Apicomplexa) or those with fragile thin-walled eggs (e.g. *Strongylus*) that may not preserve intact in coprolites [6]. Paleoparasitological analysis of extinct animal coprolites can not only inform us about host-parasite relationships, but may also be able to shed new light on biodiversity loss due to the process of coextinction where parasitic and mutualistic taxa disappear along with their host taxon. The relative importance of coextinction in total biodiversity loss is poorly understood due to a lack of empirical data [22], but analysis of coprolites may provide a way to obtain such data.

Recently, an ideal resource on which to perform a broad-scale study of gastrointestinal parasites from an extinct megafauna group has been uncovered in New Zealand. Here, accumulations of Holocene coprolites have been excavated from several cave and rock overhang sites on across the South Island [5], [23]–[24]. Identification of the coprolites using aDNA analysis has revealed they were deposited by moa (Aves: Dinornithiformes), a group of large avian herbivores that formerly occurred throughout New Zealand. Nine species of moa [25], [26], ranging from c. 30 to >200 kg [27], all underwent rapid extermination following initial settlement of New Zealand in the 13th Century AD [28]. Analyses of several of the coprolites has already provided answers to some questions regarding moa biology, including diet, habitat-use and niche partitioning [5], [23]–[24]. Here, we use both microscopic and aDNA analyses of parasites from moa (Aves: Dinornithiformes) coprolites to examine host-parasite relationships, and determine whether paleoparasitological analysis of coprolites may provide a suitable method for detecting coextinction events.

## Materials and Methods

### Coprolite Samples

**Figure 1:**
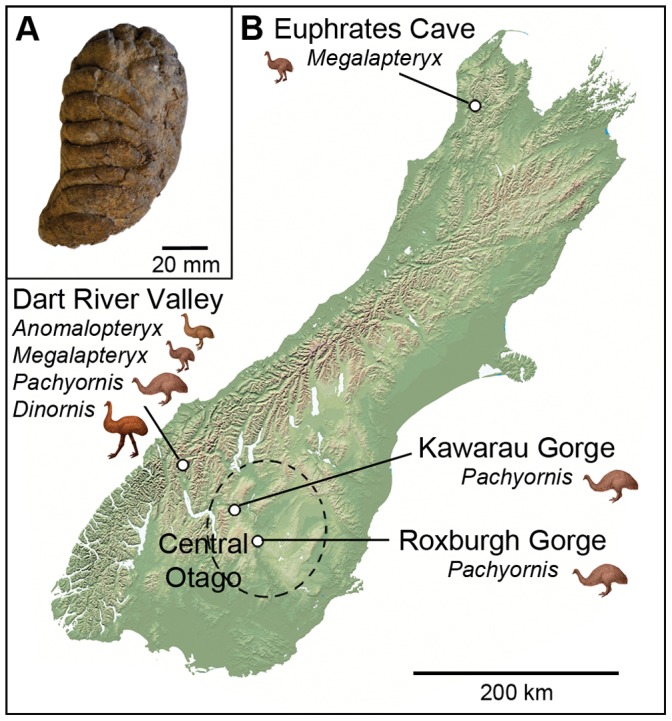
Location and taxonomic representation of moa coprolite study sites. *(a)*, Moa coprolite from Dart River Valley *(b)*, Moa coprolite sites on the South Island of New Zealand, showing moa taxa represented in coprolite assemblages.

We used coprolites for which the depositing moa species had previously been determined by aDNA analysis [5, 24, unpublished data] (Table S1). The coprolites were all intact boli and in an excellent state of preservation (figured in 5, and supplementary material of 24). We examined the presence and abundance of parasites in the coprolites (n = 84) using microscopic techniques, and used molecular analyses on a subset of the total (n = 16). The coprolites represent four moa species and three geographic regions of New Zealand’s South Island (Fig. 1): the relatively high rainfall Dart River Valley (c. 500 m elevation), in the mountains of West Otago (heavy-footed moa, *Pachyornis elephantopus*, n = 8; South Island giant moa, *Dinornis robustus*, n = 17; little bush moa, *Anomalopteryx didiformis*, n = 3; upland moa, *Megalapteryx didinus*, n = 19); the Kawarau and Roxburgh River gorges (c. 200 m elevation) in the semi-arid region of Central Otago (*P. elephantopus*, n = 2); and the subalpine Euphrates Cave (c. 1000 m elevation), located on the Garibaldi Ridge, Northwest Nelson (*M. didinus*, n = 35). The Dart River and Central Otago coprolites are of late Holocene age (<3,000 BP) [5], [29], and the Euphrates Cave coprolites extend from the late to mid Holocene (oldest dated sample 6,368±31 radiocarbon years BP) until the approximate time of moa extinction [24]. Unprocessed remainders from the coprolites have been accessioned into the collections of Canterbury Museum for permanent storage (Table S1).

### Microscopic Examination of Parasites

Subsamples (0.31–0.10 g) from each coprolite were boiled in KOH for ten minutes. A known number of exotic *Lycopodium* spores [30] (1–2 tablets, batch number 483216, mean of 18583 spores per tablet) were added to each sample. The resulting sediment was pipette mixed, and drops were mounted on microscope slides (2–3 per sample) in glycerol jelly. Slides were systematically scanned at 200×magnification, and helminth eggs and *Lycopodium* spores were counted, allowing quantification of egg abundance. Because of a change in laboratory operating procedures that occurred during this study, samples that were prepared earlier are quantified according to volume (mL), while those prepared later are quantified according to mass (g). The slides are held by Landcare Research, Lincoln, New Zealand.

### aDNA Analysis

DNA extraction, polymerase chain reaction (PCR), cloning, and sequencing were carried out following the methods of Wood et al. [24] at the Australian Centre for Ancient DNA. Ancient DNA (aDNA) extraction and PCR setup was carried out in a geographically and physically isolated dedicated aDNA laboratory located 15 minutes from the University of Adelaide campus, where downstream procedures were performed in a modern DNA laboratory. Protocols to control for contamination [31] were strictly followed, including the use of Shrimp DNase to eliminate potential contaminants in PCR reagents.

We designed two sets of primers: (1) Nem18SF (5′-ATTCCGATAACGARCGAGAC-3′) and Nem18SR (5′-CCGCTKRTCCCTCTAAGAAGT-3′); (2) Nem18SlongF (5′-CAGGGCAAGTCTGGTGCCAGCAGC-3′) and Nem18SlongR (5′-GACTTTCGTTCTTGATTAATGAA-3′). Both sets of primers bind to regions that are conserved across a broad range of invertebrates (including the common parasitic helminth groups of apicomplexans, nematodes, and trematodes), and amplify variable regions of the 18S gene (c. 40–120 bp with Nem18S primers; c. 350–400 bp with Nem18Slong primers) (Figs. S1, S2, S3, S4).

Preliminary identification of clone sequences was performed using BLAST. If sequences were obtained using both primer sets, from the same coprolite, and returned identical taxonomic matches in BLAST, then these sequences were assumed to represent the same taxa and were concatenated for the phylogenetic analysis. Sequences from the coprolites were sorted into apicomplexans, nematodes, and trematodes based on nearest BLAST matches. These were aligned (using MUSCLE in Geneious) with 18S sequences (c.1700–1900 bp) from a range of representative taxa from each of these parasite groups (Table S2). The alignments were imported into BEAUti v.1.6.1 and the resulting xml. file was analysed using BEAST v.1.6.1. Our analyses incorporated a HKY model with estimated base frequencies and an age-independent transitions only sequence error model, a relaxed lognormal clock and a Yule tree prior (lognormal birth rate). Maximum credibility trees were produced from MCMC chain lengths of 25 (Apicomplexa, Trematoda) or 50 (Nematoda) million generations (parameters logged every 1000) and assessed for robustness using Tracer v.1.5. Tree output files were summarized using Tree Annotator (10% burnin). Sequences >50 bp in length were deposited in GenBank (Accession numbers KC405320– KC405484).

## Results

### Microscopic Examination of Parasites

Nematode eggs were observed on microscope slides, although no larvae were seen. The taxonomic usefulness of many helminth eggs is limited and identification can prove difficult [6], [32]. Here we describe the egg types present in the moa coprolites and suggest their likely taxonomic affinities. Prevalence and abundance of the different nematode egg types are shown in Table 1. Coprolites from low altitude sites (Dart River Valley, Kawarau Gorge and Roxburgh Gorge) had higher parasite egg diversity and prevalence overall (mean egg types per moa species = 2.25; eggs in 51% of coprolites) compared with those from the higher altitude Euphrates Cave (mean egg types per moa species = 1; eggs in 17.1% of coprolites). It was not possible to test whether these patterns were significant overall, due to two methods having been used to quantify egg abundance (eggs mL^−1^ and eggs g^−1^). However, for coprolites where egg abundance was calculated as eggs g^−1^, there was a significantly lower prevalence at Euphrates Cave (1000 m elevation) compared with Dart River (500 m elevation) (p = 0.037, t-test).

**Table 1:** Prevalence and abundance of nematode egg types in moa coprolites.

Locality and moa species	n	Egg type 1 cf. Heterakoidea			Egg type 2 undetermined Nematoda			Egg type 3 cf. Trichinellidae			
		P	A_m_	A_r_	P	A_m_	A_r_	P	A_m_	A_r_	
**Dart River Valley**											
*Anomalopteryx didiformis*	**3**	**0.67**	**876**	**370–1383**	**0**	**0**	**0**	**0**	**0**	**0**	
*Dinornis robustus*	6	0.5	960	245–2203	0	0	0	0	0	0	
	11	0.55	1148*	347–1931*	0.09	7288*	0–7288*	0	0	0	
	**17**	**0.53**			**0.06**						
*Pachyornis elephantopus*	5	0.2	2655	0–2655	0	0	0	0.2	490	0–490	
	3	0.67	1636*	1101–2170*	0.33	271*	0–271*	0	0	0	
	**8**	**0.38**			**0.13**			**0.13**			
*Megalapteryx didinus*	15	0.2	363	226–570	0	0	0	0.13	280	262–298	
	4	0.25	307*	0–307*	0.5	303*	262–344*	0	0	0	
	**19**	**0.21**			**0.11**			**0.11**			
**Kawarau Gorge**											
*Pachyornis elephantopus*	1	1.0	4645*	–	1.0	3650*	–	0	0	0	
											
**Roxburgh Gorge**											
*Pachyornis elephantopus*	1	0	0	0	0	0	0	0	0	0	
											
**Euphrates Cave**											
*Megalapteryx didinus*	35	0	0	0	0	0	0	0.17	653	241–965	

P = prevalence (proportion of coprolites in which the egg type was present), A_m_ = mean abundance where present, A_r_ = range of abundance where present. A_m_ and A_r_ are shown as eggs g^−1^, except where a * symbol signifies the data are eggs mL^−1^.

#### Egg type 1 (Fig. 2a)

*Description*: Ovoid to slightly barrel-shaped (approximately parallel sides). Egg wall relatively thick (c. 5–6 µm), transparent, equal thickness around entire egg, with smooth outer surface. Inner mass often golden colour and shrunken away from outer wall. *Dimensions*: 50–67.5×31.5–45 µm (mean 61×39 µm) (n = 16). *Likely affinity*: Eggs are similar to those of Heterakoidea spp. (Nematoda). *Hosts*: *A. didiformis*, *D. robustus*, *P. elephantopus*, *M. didinus*. Present in coprolites from the Dart River Valley and Kawarau Gorge.

**Figure 2:**
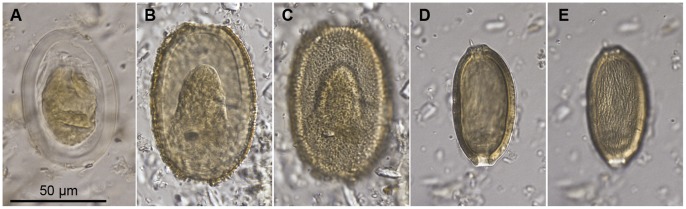
Helminth eggs from moa coprolites. *(a)*, egg type 1, cf. Heterakoidea; *(b–c)*, egg type 2, undetermined Nematoda; *(d–e)*, egg type 3, Trichinellidae cf. *Capillaria*.

#### Egg type 2 (Fig. 2b, c)

*Description*: Ovoid, light brown. Wall c. 5 µm thick. Outer layer with short, evenly spaced spinules protruding up to 3 µm from the surface. The spinulose layer is often absent around the poles. *Dimensions*: 70–72.5×40–47.5 µm (n = 4). *Likely affinity*: undetermined Nematoda. Appears to be similar to egg type 1 but with a spinulose layer on the external surface. *Hosts*: *D. robustus*, *P. elephantopus*, *M. didinus*. Present in coprolites from the Dart River Valley and Kawarau Gorge.

#### Egg type 3 (Fig. 2d, e)

*Description*: Elongate ovoid, orange-brown, surface grades from reticulate to longitudinally striate. Obvious polar pores. *Dimensions*: 52–60×30–35 µm (n = 2). *Likely affinity*: Trichinellidae (c.f. *Capillaria*) (Nematoda), due to the presence of polar pores. *Hosts*: *P. elephantopus* and *M. didinus*. Present in coprolites from the Dart River Valley and Euphrates Cave.

### aDNA Analysis

In total, 167/233 (71.7%) of the clone sequences using the Nem18S primers and 42/61 (68.9%) of the clone sequences using the Nem18Slong primers were identified as being from parasites. Six distinct clusters of parasite clone sequences, likely to represent identical or very closely related taxa, were identified from sequence alignments and BLAST matches (Figs. S5, S6). Three of the groups had nearest BLAST matches within Apicomplexa, two within Nematoda and one within Trematoda. Non-target sequences included moa, plant, fungi and soil micro-organisms. Fish sequences obtained in initial PCRs were eliminated by using shrimp DNAase, and therefore likely reflected contaminants in the PCR reagents.

#### Maximum credibility trees

Single representative sequences from each of the six groups were used in this analysis, and are listed in Table S3.

### Apicomplexa

**Figure 3:**
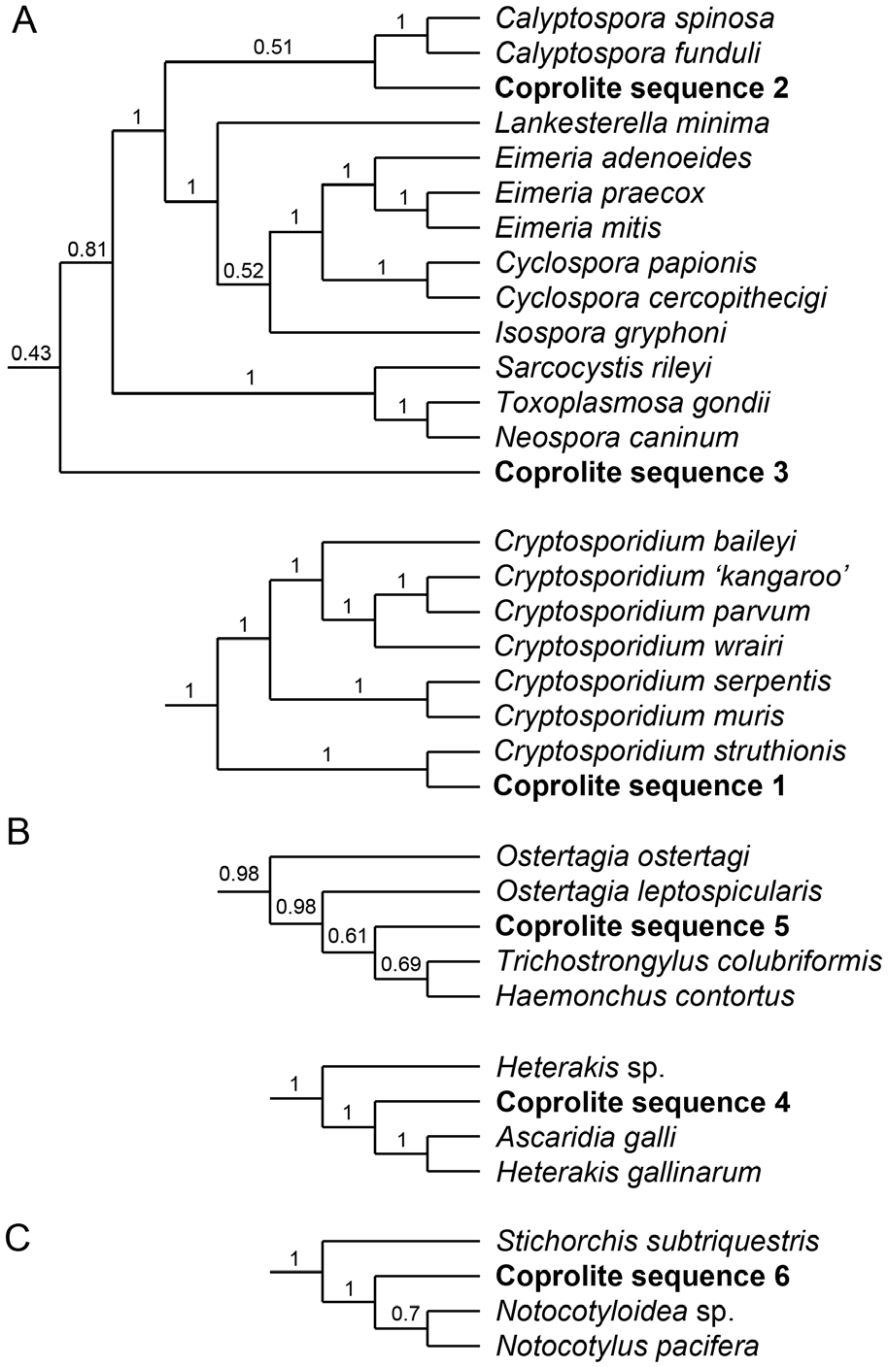
Phylogenetic position of 18S sequence groups obtained from moa coprolites. *(a)*, apicomplexa; *(b)*, nematoda; *(c)*, trematoda. The complete maximum credibility trees are provided as Figs. S7, S8, S9.

Sequence 1, obtained from a single Dart River *M. didinus* coprolite (Table 2), was well-supported (posterior value 1.0) within the clade including *Cryptosporidium* species, and sister (100% bootstrap support) to *C*. ‘*struthionis*’, an undescribed strain sequenced from ostrich (*Struthio camelus*) (Figs. 3, S7). Sequences 2 (*M. didinus* from Dart River and Euphrates Cave) and 3 (*D. robustus* from Dart River) were both placed within the suborder Eimeriorina with posterior values of 1.0 and 0.43 respectively (sequence 2 as sister to Calyptosporidae and 3 as sister to all non-Cryptosporidiidae Eimeriorina) (Fig. S7). It should be noted that several families within Eimeriorina (Elleipsisomatidae, Selenococcidiidae, and Spirocystidae) were not represented in the analysis due to unavailability of 18S sequences on Genbank, and this may have affected the exact placement of clones within this group.

**Table 2:** Prevalence of six helminth taxa identified by aDNA analysis of moa coprolites.

		Dart River Valley				Euphrates Cave
		*Anomalopteryx* *didiformis*	*Dinornis robustus*	*Pachyornis elephantopus*	*Megalapteryx* *didinus*	*Megalapteryx didinus*
	**Coprolites analysed (n)**	1	3	2	4	6
	**Minimum individual birds** #	1	2	2	4	6
	**Total clones** *	22/0	27/21	14/0	95/40	75/0
**Sequence** **group**	**Identity**					
1	*Cryptosporidium*	–	–	–	0.25	–
2	Eimeriorina sp. 1	–	–	–	1.0	0.17
3	Eimeriorina sp. 2	–	0.33	–	–	–
4	Heterakoidea	1.0	1.0	0.5	0.5	0.17
5	Trichostrongylidae	–	–	–	–	0.17
6	Echinostomida	–	–	–	1.0	0.67

# minimum individual moa represented by analysed coprolites based on moa haplotypes and radiocarbon dates;

* number of clones obtained using Nem18S primers/Nem18Slong primers.

### Nematoda

Both Nematoda sequences were placed within well-supported clades (Fig. S8). Sequence 4 is nested within the superfamily Heterakoidea (posterior value 1.0) (Figs. 3, S8), and was the most widespread sequence obtained, occurring in coprolites from all four moa species and both Dart River Valley and Euphrates Cave (Table 2). Sequence 5 is nested within the family Trichostrongylidae (posterior value 0.98) (Figs. 3, S8) and was recorded from just a single *M. didinus* coprolite from Euphrates Cave (Table 2).

### Trematoda

Sequence 6 (*M. didinus* from Dart River and Euphrates Cave; Table 2) is well-supported as being nested within the Trematode order Echinostomida (posterior value 1.0), and sister to Notocotylidae (Figs. 3, S9).

## Discussion

### Parasite Assemblage

The parasite groups identified from the moa coprolites have all been recorded previously from New Zealand birds [33] and are typical of parasites recorded from extant ratites [34]–[35]. Although there was some overlap in the parasite taxa identified by both microscopic and DNA analysis (Heterakoidea), each analysis method detected taxa that the other did not, highlighting the usefulness of a multidisciplinary approach in paleoparasitological studies. Whereas microscopic analysis was used on more samples and may have detected some of the less prevalent parasite taxa, DNA analysis detected coccidians, which due to their small size may not have preserved as well as larger, thick-walled eggs.

Within each of the six parasite DNA sequence groups (Figs. S5, S6) there is some genetic variation, which may partly be due to ancient DNA damage [36], but may also represent different parasite haplotypes or closely-related species. An example of the latter is evident in group 6 (Echinostomida), where sequences with an A in position 99 were recovered from both Dart River Valley and Euphrates Cave coprolites, but sequences with a G in that position were present only in coprolites from Euphrates Cave (Fig. S5).

Our results for nematode egg counts (Table 1) indicate that moa at lower altitudes had higher parasite prevalence and diversity. This does not appear to be due to a higher number of moa species represented in the lowland coprolite assemblages. For example, all three nematode egg types were detected in *M. didinus* coprolites from Dart River Valley (overall prevalence 0.31), whereas just one type was present in the *M. didinus* coprolites from Euphrates Cave (overall prevalence 0.17). This effect is likely due to host density, with has been shown to positively correlate with parasite abundance [37]. In prehuman New Zealand, moa density was probably relatively high in lowland sites, where multiple sympatric species often coexisted (i.e. remains of four moa species from the Dart River Valley, one from Euphrates Cave). However, the lower parasite prevalence observed at Euphrates Cave may also be partly due to the extended temporal range of the coprolites from the site. Whereas coprolites from the low altitude sites are all late Holocene (a period of relative climatic stability), coprolites from Euphrates Cave extend back into the early-mid Holocene period, when climatic conditions in that region were relatively warm and dry [38]. A larger sample of radiocarbon dated coprolites from Euphrates Cave would be required to test any potential temporal affect on parasite prevalence.

### Gondwanan Vicariance

The hypothesis that New Zealand’s indigenous fauna and flora represent recent dispersal events following complete submergence of the current landmass during the Oligocene has been a topic of contentious debate during the last decade [39], [40]. Moa have long been regarded as having vicariant Gondwanan origins [41], although recent molecular studies using mitochondrial DNA have presented a range of widely varying taxonomic relationships and evolutionary histories for palaeognaths, some inferring repeated dispersal events and loss of flight (reviewed by Allentoft and Rawlence [42]). However, a recent re-evaluation of morphological relationships suggests that a vicariant Gondwanan origin for moa cannot be ruled out [43]. This is in line with new fossil evidence providing support for the presence of multiple ancient vicariant lineages in New Zealand, including sphenodontine reptiles [44], acanthisittid wrens [45] and freshwater limpets (*Latia*) [46]. Our results provide some evidence that moa parasites may also have Gondwanan affinities.

Within the Heterakoidea, Inglis & Harris [47] erected the family Kiwinematidae to include the genera *Hatterianema* and *Kiwinema* described from New Zealand’s indigenous tuatara (*Sphenodon punctatus*) and kiwi (*Apteryx* sp.) respectively. Subsequently, a newly erected African genus *Mammalakis* has also been placed within Kiwinematidae [48]. Kiwinematidae are characterized by primitive features from which features in other Heterakoidea families could have hypothetically been derived, and thus may represent a Gondwanan relict [47]. Although we cannot definitely attribute the Heterakoidea from moa coprolites to Kiwinematidae because this family is not represented on Genbank, the DNA sequence is sufficiently divergent from both *Ascaridia* and *Heterakis* to suggest that it belongs to neither of these widespread genera (pairwise % identities for Nem18Slong fragment: 97.4% *Ascaridia galli* : moa taxon; 95.9% *Heterakis gallinarum* : moa taxon; 98.4% *Ascaridia galli* : *Heterakis gallinarum*). Further DNA work on extant Kiwinematidae may help resolve the placement of the moa coprolite taxon within Heterakoidea.

Another potential Gondwanan link may lie in the *Cryptosporidium* sequenced from a moa coprolite. Of seven *Cryptosporidium* spp. included in the phylogenetic analysis, the coprolite sequence formed a well-supported ‘ratite’ clade with *Cryptosporidium* ‘*struthionis*’, basal to the two *Cryptosporidium* clades reported by Xiao et al. [49] from mammals, snakes and lizards (Figs. 3, S7). A diverse array of avian *Cryptosporidium* genotypes have been recognized using the SSU rRNA locus [50], and future phylogenetic analyses of this parasite group may provide an interesting complimentary data set with which to understand evolutionary relationships between bird groups.

### Coextinction and Habitat Fragmentation

The process of coextinction (loss of parasitic and mutualistic taxa) makes a significant contribution to biodiversity loss during extinction events [51], and could potentially account for the majority of species losses [52]. Therefore, an understanding of the process could assist with making more accurate estimates of the total numbers of species at risk of extinction [51]–[52]. A lack of empirical data has so far precluded an accurate assessment of the importance of coextinction in overall biodiversity loss [52]. Paleoparasitological analysis of coprolites from extinct animals such as moa may provide important quantitative case studies of coextinction events, at least of gastrointestinal parasites.

**Figure 4:**
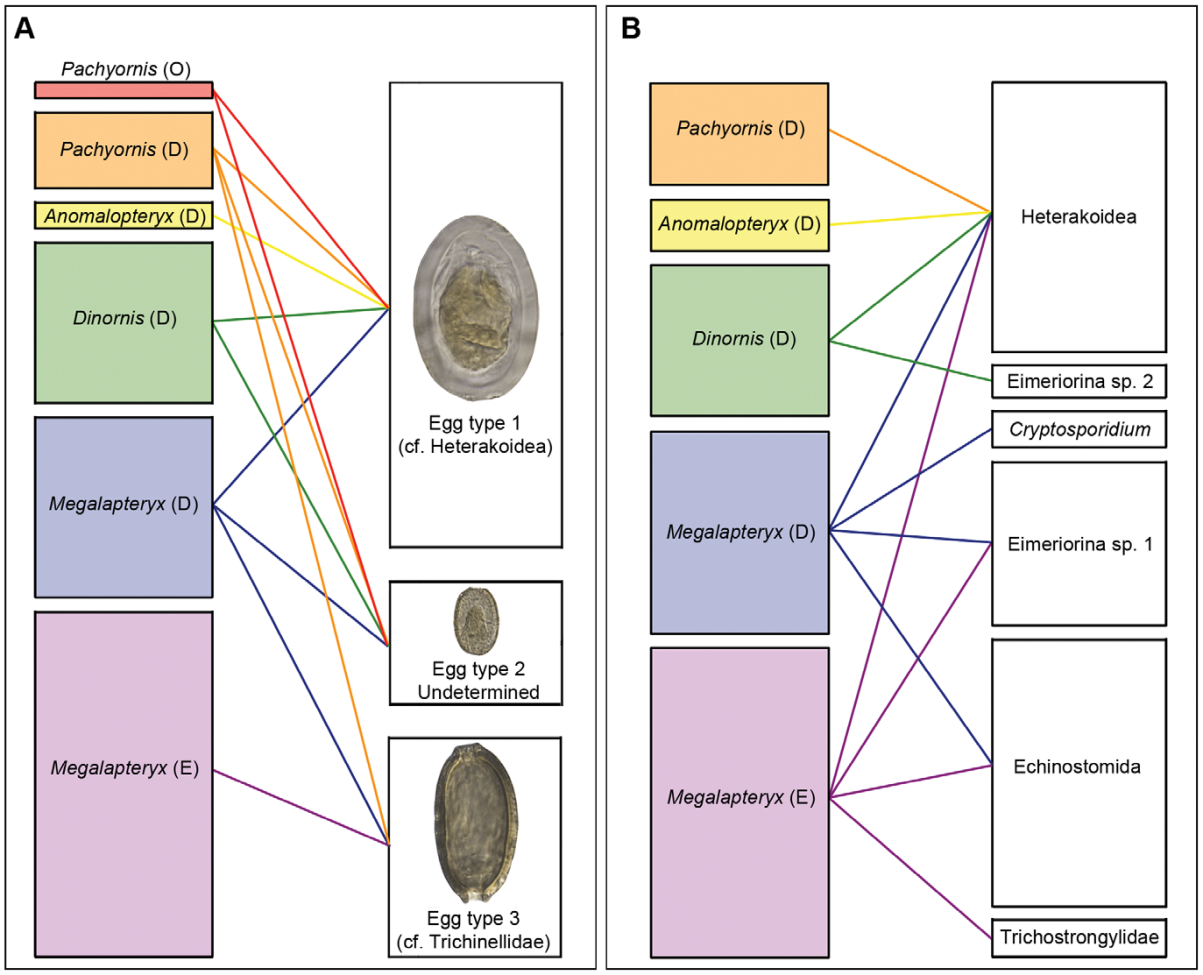
Network diagrams of identified moa - parasite interactions. *(a)* interactions between moa species and parasite egg types, based on morphological identification of parasite eggs from 84 coprolites; *(b)* interactions between moa species and DNA sequence groups, based on DNA identifications of parasites from a subset of 16 coprolites. Parasite boxes are scaled relative to overall prevalence in analysed coprolite assemblages and moa bones are proportional to the number of coprolites analysed from each species/locality. Localities (in parentheses) are: O, Central Otago; D, Dart River Valley; E, Euphrates Cave.

The question of whether parasite coextinctions occurred in New Zealand is not new; in 1994, Bush and Kennedy [53] pondered whether “when the moas of New Zealand went extinct over a century ago, did they take with them parasite metapopulations or are those parasites found today in other ratites (e.g. the kiwi, emu, cassowary, rhea and ostrich)?”. Although a complete 18S DNA survey of gastrointestinal parasites in extant New Zealand birds would be required to prove whether the taxa present in moa coprolites are now extinct, some of the taxa were identified only from coprolites of *Megalapteryx*, providing some evidence for host-specificity and likely coextinction (Fig. 4). These taxa include species in the order Echinostomida and suborder Eimeriorina, which were both present in all *Megalapteryx* coprolites from Dart River and in coprolites from Euphrates Cave, but were not present in coprolites of the three other moa species (Table 2). The Echinostomida sequence was in a well-supported clade with members of the Notocotylidae (*Catatropis* and *Notocotylus*), a family of trematodes that inhabit the digestive tracts (commonly caeca) of mammals and birds [54]. Both *Catatropis* and *Notocotylus* have been recorded from wild birds in New Zealand, in particular avian herbivores in close association with water (ducks, geese) and wading birds [33]. Aquatic snails, such as the native *Potamopyrgus antipodarum* are the intermediate hosts [55]. The presence of such a parasite in *Megalapteryx* would not be unexpected, as evidence from coprolites show that this moa species occasionally fed around margins of alpine tarns and lakes, and grazed aquatic plants [56]. Eimeriorina sp. 2, Trichostrongylidae and *Cryptosporidium* were also only identified from *Megalapteryx*, but from single coprolites, so further samples would need to be analysed to test the host-specificity of these taxa.

The apparent differences in parasite diversity between moa species (Tables 1, 2) could be due to several factors. The evolutionary histories of hosts and parasites are often closely mirrored [57], and the basal position of *Megalapteryx* within moa [25] may explain the apparent host specificity of several parasites identified from coprolites of this species. However *Dinornis* is the second most basal moa genus, yet we found similar parasite diversity to *Pachyornis* and *Anomalopteryx*, which represent more recent splits within the moa phylogeny [25]. Another possibility is that the parasite diversity may relate to the species’ ecology. For example, *Megalapteryx* may have favoured feeding near water sources such as small alpine tarns [56] and therefore been more susceptible to waterborne parasites (e.g., *Cryptosporidium*) and parasites with aquatic intermediate hosts.

Analysis of parasites in coprolites from other New Zealand extant bird species offers the potential to examine how parasite communities deal with severe habitat fragmentation. For example, the extensive pre-European kakapo (*Strigops habroptilus*) coprolite record across New Zealand [58], [59] could provide a means to contrast past kakapo parasite diversity with that in modern birds, which now have severely contracted population size (<150 individual birds) and distribution (few offshore islands).

### Conclusions

We have confirmed the presence of apicomplexan, nematode and trematode gastrointestinal parasites in the coprolites of New Zealand’s extinct moa. Several of these parasites appear to have been host-specific, and therefore are likely to have become extinct with the moa. This study has shown the potential for multidisciplinary paleoparasitological studies of coprolites to contribute to our understanding of evolutionary histories of both parasites and hosts, and to provide case studies of coextinction. The relatively young ages of moa coprolites, and the recent discovery of many specimens from multiple sites, offer an ideal sample for such a study. Questions relating to parasite-host evolutionary histories, and coextinction, could also be answered by detailed paleoparasitological analyses of many Late Quaternary coprolites known from around the world, including ground sloth [9] and mammoth [60].

## Supporting Information

Figure S1 **Alignment of Nematoda, Trematoda, and Apicomplexa 18S sequences used for designing the Nem18SF and Nem18SR primers.**
(DOC)

Figure S2 **Alignment of Nematoda 18S sequences used for designing the Nem18SlongF and Nem18SlongR primers.**
(DOC)

FigureS3 **Alignment of Trematoda 18S sequences for designing the Nem18SlongF and Nem18SlongR primers.**
(DOC)

Figure S4 **Alignment of Apicomplexa 18S sequences for designing the Nem18SlongF and Nem18SlongR primers.**
(DOC)

Figure S5 **Alignment of clone sequences obtained from moa coprolites using Nem18SF and Nem18SR primers.**
(DOC)

Figure S6 **Alignment of clone sequences obtained from moa coprolites using Nem18SlongF and Nem18SlongR primers.**
(DOC)

Figure S7 **Maximum-credibility tree for 18S sequences of representative Apicomplexa (from Genbank), and moa coprolite sequences 1–3. The tree is rooted with** ***Gymnodium*** **(Dinoflagellata).**
(JPG)

Figure S8 **Maximum-credibility tree for 18S sequences of representative Nematoda (from Genbank), and moa coprolite sequences 4–5.** The tree is rooted with *Gordius* (Nematomorpha). All sequences are correctly resolved within clades representing 5 major orders of parasitic nematodes, except *Litomosoides (Spiruda) and *Rondonia* (Rhabditida).
(JPG)

Figure S9 **Maximum-credibility tree for 18S sequences of representative Trematoda (from Genbank), and moa coprolite sequence 6.** The tree is rooted with *Notocaryoplana* (Turbullaria).
(JPG)

Table S1 **Moa coprolite specimens that were used in this study.** Specimen numbers relate to the Australian Centre for Ancient DNA sample database (A). Where voucher specimens exist, the museum registration numbers are also given (CM, Canterbury Museum, New Zealand; OM, Otago Museum, New Zealand).
(DOC)

Table S2 **18S sequences from GenBank that were used in the phylogenetic analyses.**
(DOC)

Table S3 **Six distinct parasite sequences obtained from moa coprolites that were used in the phylogenetic analyses.** Note that the analyses of sequences 1, 2 and 4 used the concatenated sequences from both primer sets (see methods section for explanation).
(DOC)
