# Supplementary material for: A Megafauna’s Microfauna: Gastrointestinal Parasites of New Zealand’s Extinct Moa (Aves: Dinornithiformes)
Source: PLoS One. 2013 Feb 25;8(2):e57315. doi: 10.1371/journal.pone.0057315 (PMC3581471; doi:10.1371/journal.pone.0057315)
Supplement: Figure S1 — Alignment of Nematoda, Trematoda, and Apicomplexa 18S sequences used for designing the Nem18SF and Nem18SR primers. (DOC) [file pone.0057315.s001.doc]

**Nem18SF Nem18SR**

**ATTCCGATAACGARCGAGACTC> <ACTTCTTAGAGGGAYMAGCGG**

NEMATODA

*Ancylostoma* ATTCCGATAACGAGCGAGACTCTAGCCTGCTAAATAGTGGCTGGATTTT----TACGTCCAGTCTACTTCTTAGAGGGATAAGCGG

*Heterakis* ............................A..........T.......A.T.TTCTT...T..A.G..............C......

*Ascaradia* .......................GA...A..........T..A....A....T.TT...TT.A.G..............C......

*Capillaria* ..C..........A.........G....A....C.....A.G.CG.GG.C.TT.T..C.G..C--..............CC.....

Eucoleus ..C..........A.........G....A....C.....A.G.CG.GG.C....TTTCG.T.AGC..............CC.....

*Ascaris* ............................AT........CATC....AAA....C.A....G.AAG..............C......

*Trichostrongylus* .......................................................A..............................

*Toxocara* ............................A.........CATC....AAA....C.A....G.AAG..............C......

*Porrocaecum* ............................A.........C.TC....AAA-...C.G....G.AAG..............C......

*Cyrnea* ............................A.........TA......A.CCCAT.TGA.....ATA..............C......

*Amidostomum* ................................................A.....................................

*Angiostrongylus* .......................................A..A....A......GT...T..........................

*Chabertia* ......................................................................................

*Cylicocyclus* ......................................................................................

*Deletrocephalus* .......................................................T..............................

*Hypodontus* ................................................C......G..............................

*Metastrongylus* .......................................A..A................T..........................

*Petrovinema* ......................................................................................

*Protostrongylus* .......................................A..A....C...........T..........................

*Stephanurus* .......................................................T..............................

*Syngamus* .......................................R..............T...............................

*Zoniolaimus* ................................................C.....TG..............................

**Nem18SF Nem18SR**

**ATTCCGATAACGARCGAGACTC> <ACTTCTTAGAGGGAYMAGCGG**

TREMATODA

Consensus ATTCCGATAACGAACGAGACTTTGGCCTGCTAAATAGTATGCCTGT-CCTCTGTGCTCGTGCAGGTGGCGGTGTTC-AYTGCCTC-YTCGTGGGGTG---GTGGTGTCGTTGGCCGGCGGGTGCGGCGCAGGTGTTTACTTCTTAGAGGGACAAGCGG

*Notocaryoplana arctica* ..............................................-...............G.ACT.........-.CC..T..-T...G.A..G.----.....C...CT.T.....CTGA.T.G.......C...T..T.....C..........

*Transversotrema haasi* .....................C..................A.A...-..C...CA...........TTT.TCA...-TCA...CGTT..AC..AC..----.....G.A..AT........C....T...T..AA....................T.A

*Trichobilharzia regenti* .......................AA...A..........GATTG..-.............T....GC....CT.CT-.T...T..-T.TA...A..A---...TG.....GAT.........A...T..CA..T...................GA...

*Rugogaster hydrolagi* ..............................................-......C........G...C.......C.C.T......TT..CG......---A..TC.C...................................................

*Notocotylus pacifera* .......................T......................-.............T.....CA......AA-GC......-C.T.......A---....A.......T...A................AA.......................

*Echinostoma paraensei* ..............................................-...................TA..T...C.-.T......-C.........A---.....TG.....A....................AC......................A

*Dicrogaster contracta* .......................................CT.....-...T...........G..C......TC..-.T......-T...G...T-.---A....A.....C..........A.T.........CC......................

*Aponurus* sp. ......................................GA.T.C..-.T........T.CTA..AG.CT...TCG.-TAC.ATCT-T..AAA..T..A--AGT..CCA...TT.T...TT......T..G.T-T..C................GA...

*Saturnius* sp. .......................A.........C.........C..-.T......A.G..A.G..G.TAC..T.GT-.G..ATC.-C.T.A...T..---T.AC.TCGAA.CCT....T.CCA...T..G...AG.......................

*Robinia aurata* .......................T..............GCTT.C..-.T........T..T...AG..TCAA.A.T-.G.AATC.-T.......T.AC--.G.T.T.G..CTT.T.A.T...C...T..G..-.G.......................

**Nem18SF Nem18SR**

**ATTCCGATAACGARCGAGACTC> <ACTTCTTAGAGGGAYMA-GCGG**

APICOMPLEXA

Consensus ATTCCGTTAACGAACGAGACCTTAACCTGCTAAATAGGGTC----------------------AGTAAC------NNTTTTTGTTCTTGTATCACTTCTTAGAGGGACTTTGCGT

*Aggregata octopiana* ....................T....TA.AT...G...A.A-----------------------.A...TGTATGATA.A...T.A...T.GATGA..T..T..T...T...ATA.

*Hepatozoon ayorgborgi* ........................................T----------------------.AA...---------.......T..AA..T..........A...........

*Calyptospora spinosa* ...T.....................................GTCAAGTGTTTTGAAGGTATAT.....AATATATAT....GA..GAC...........................

*Cryptosporidium serpentis* .....................................ATAA----------------------TAA..A-----TTTA....T..A..T...--.....................

*Neospora caninum* ......................................A..----------------------..G...--------..CG..................................

*Lankesterella minima* ...T..A.............-...G.............A..----------------------T.G...-------G..A.A....CA.C.........................

*Eimeria mitis* ...T..A................GG................----------------------G..G..CCTGGGTCACCAG..CACC.C...G.....................
