# Supplementary material for: A Megafauna’s Microfauna: Gastrointestinal Parasites of New Zealand’s Extinct Moa (Aves: Dinornithiformes)
Source: PLoS One. 2013 Feb 25;8(2):e57315. doi: 10.1371/journal.pone.0057315 (PMC3581471; doi:10.1371/journal.pone.0057315)
Supplement: Figure S2 — Alignment of Nematoda 18S sequences used for designing the Nem18SlongF and Nem18SlongR primers. (DOC) [file pone.0057315.s002.doc]

**Nem18SlongF**

**CAGGGCAAGTCTGGTGCCAGCAGC>**

NEMATODA

Consensus GAGGGCAAGTCTGGTGCCAGCAGCCGCGGTAATTCCAGCTCTCAAAGTGTATATCGTCATTGCTGCGGTTAAAAAGCTCGTAGTTGGATCTGCGCCTCAG

*Heterakis* .........................................C.....................................A.......G..AT...TA...

*Ascaridia* .....................................................................................T...A.....TA...

*Ascaris* ....................................................................................................

*Wellcomia* ...R......................................T.T............T.C.................................ATTCA..

*Oxyuris* ..........................................................................................C.A..T.G..

*Toxocara* ....................................................................................................

*Terranova* ....................................................................................................

*Dranunculus* .........C......................C........................T..............................AT.CA..T.G.C

*Anisakis* ....................................................................................................

Consensus GACTTGGTCCGCCCA-CTGGGCGA-GAACTGGGCTC--CT--GGGCTAG--------TACTGCTGGTTTTCCC-------TACGTTGCCTTCATCGGTCG

*Heterakis* ..T.C......T..T-A.......-......AA...--..--......A--------................-------....................

*Ascaridi*a ..T.C..........-T......T-......AA...--..--.......--------..T..T.........T-------......A....A......T.

*Ascaris* ...C...........-........-...........--..--.......--------.T..............-------....................

*Wellcomia* CT........A..A.TT.T..T.TT....CAA.T.TATT.TT..CT.GAACAATAAT..A..........TTTTTTGCTA..G...A....T..T.....

*Oxyuris* ..........A..A-TT.C..T.CT......A....CT.----A....---GT---TATG..........-------CCT...........G.....C..

*Toxocara* ...............-........-...........--..--.......--------.T..............-------....................

*Terranova* ...C...........-........-...........--..--.......--------.T.G..........T.-------.............C......

*Dranunculus* ...A......ATT.-AT...AT.T-.....AT.A-TCGTA---.....ATC-------TA..T.........-------T.G....A....G........

*Anisakis* ...............-........-...........--..--.......--------................-------....................

Consensus CGTAGGGTGGCTAGCGAGTTTACTTTGAAAAAATTAGAGTGCTTAACGCGGGCTT--A-TGCCTGAATAYTCGTGCATGGAATAATAGAATAGGATCTCG

*Heterakis* ......................................................A--T-..........T..............................

*Ascaridia* ....A....A...A.........................................--.-..........T................G.............

*Ascaris* ............................................C..........--.-..T.......C..............................

*Wellcomia* .C..TA......G..AG................G..A....A....A..A....ATTTA..........A.G..............GA............

*Oxyuris* ..............................................A..A...G.TC.-G-........T.T............................

*Toxocara* ............................................C..........--.-..T.......C..............................

*Terranova* ............................................CG.........--.-..T.......C..............................

*Dranunculus* ....A..................C......................T........-A.AA.........T..............................

*Anisaki*s ............................................C..........--.-..T.......C..............................

Consensus GTTCTATTTTGTT-GGTTTTCTGATCTGAGATAATGGTTAAGAGGGACGGACGGGGGCATTCGTATCGCTGCGTGAGAGGTGAAATTCTTGGACCGTAGC

*Heterakis* .............-......................................................................................

*Ascaridia* .............-..................................A.....................T.............................

*Ascaris* .............-......................................................................................

*Wellcomia* A.........-G.T..........AT..........A...........AA.......................C...............G..........

*Oxyuris* .C........-G.T.........G..C.....................A...................................................

*Toxocara* .............-......................................................................................

*Terranova* .C...........-......................................................................................

*Dranunculu*s .............-..................................A................C..................................

*Anisakis* .............-......................................................................................

Nem18SlongR

<TTCATTAATCAAGAACGAAAGTC

Consensus GAGACGYCCGACTGCGAAAGCATTTGCCAAGAATGTCTTCATTAATCAAGAACGAAAGTC

*Heterakis* ......G.....................................................

*Ascaridia* ......C.....................................................

*Ascaris* ......T...........T.........................................

*Wellcomis* ......C..A..........................T.......................

*Oxyuris* ......C.....................................................

*Toxocara* ......T.....................................................

*Terranova* ......C.....................................................

*Dranunculus* ......T..C..................................................

*Anisakis* ......T.....................................................
