# Supplementary material for: A Megafauna’s Microfauna: Gastrointestinal Parasites of New Zealand’s Extinct Moa (Aves: Dinornithiformes)
Source: PLoS One. 2013 Feb 25;8(2):e57315. doi: 10.1371/journal.pone.0057315 (PMC3581471; doi:10.1371/journal.pone.0057315)
Supplement: FigureS3 — Alignment of Trematoda 18S sequences for designing the Nem18SlongF and Nem18SlongR primers. (DOC) [file pone.0057315.s003.doc]

**Nem18SlongF**

**CAGGGCAAGTCTGGTGCCAGCAGC>**

**TREMATODA**

Consensus GAGGGCAAGTCTGGTGCCAGCAGCCGCGGTAACTCCAGCTCCAGAAGCGTATATTAAAGTTGTTGCAGTTAAAAAGCTCGTAGTTGGATCTGGGTCGCAT

*Transversotrema* ............................................T...................................................A...

*Robinia* ....................................................................................................

*Trichobilharzia* ...........................................A..................C.......................A.C......T.TGC

*Rugogaster* ...........................................AT.................C............................C..CAA...

*Liolope* ...........................................A..................C...................................G.

*Notocotylus* ....................................................................................................

*Echinostoma* ....................................................................................................

*Dicrogaster* ....................................................................................................

Consensus GGCTACATGCCGTTGCTTGTWYTCCTGGTCTGGTT-ACGACCGGGTCGGGTGTRY-GGGTCGGTGTAGTGGTTGTGCAGCCTTTCTGCCGTGTCTGTTTT

*Transversotrema* ......T.A..AG...CAAAGC.GT.C..GC..C.-T.TG..AT.-..TTC.GATA..TA................TG.T..................A.

*Robinia* ......GCCT...G.TA...--------.GCAA..GT...A.A.A--C.T.CAACAATCCT.AG..G.....C...T.A....................C

*Trichobilharzia* ..TCG...............TCG.G-.T.T.....-....T.A.AA.TT....TC-..C..........................A.....A.....G.A

*Rugogaster* ....G.C.AT.......CC.TCCG....C......-....T....C.C....GGTG......A.TGG..........G.......A...........GAA

*Liolope* .AT.G........C...C..TC.....AC......-G..G.......T.....TC-..................C..........A..............

*Notocotylu*s ..........T.......A.AT..TCA.C......-....T.....T....T.AT-..C.G..C....................................

*Echinostoma* .............C...C..TGGTG..........-........A..AA.CC.GC-.A......C............TT.........T..........-

*Dicrogaster* A.TC..GC.T.....G..ACATG.TC...T.....-.....TA..-.CA....GC-.AC...A...T................................C

Consensus --ACAGGTGCTGGCT-GGATYGGTGGGCTTGCCT-GCCGG---TCTGTTGGCATGCTTCCGGATGCCTTTAAACGGGTGTCGGGGGCGGACGGCACGTTT

*Transversotrema* ---........AAT--CATCT..CA...G.T...-.T.A.---.TG.......C..C...T.G.......C............A...........A....

*Robinia* -G.............----------------------------..G.C.A........T...G.........C........C..A..A.......T....

*Trichobilharzia* -A..G.......A.GGA...G....A.T.CTT..T...C.CTA.............................C...........A..........TC...

*Rugogaster* -A..G..........-..GCT..........T.CT.....---C.C..CA.........TT.........T.C........A..................

*Liolope* AA..G..........-..G.C....A......T.-.T...---C....C...................................A.........G.T...

*Notocotylus* --G..........T--T...C.T.-----------TT...---..G.C............A.......................A..........T....

*Echinostoma* --.......TCA..G-T..CT.............-..T..---................TT.G....................A.......A........

*Dicrogaster* -G.............-....C......G..T...T..T.A---.TC..............A.......................................

Consensus ACTTTGAACAAATTTGAGTGCTCAAAGCAGGCCTTTGTGCCTGAAAATTCTTGCATGGAATAATGGAATAGGACTTCGGTTCTATTTTGTTGGTTTTCGG

*Transversotrema* .............C....................AA..........G.C.G.................................................

*Robinia* .................................G.A...........G.................A..................................

*Trichobilharzia* .........................G................AG.....................A..................................

*Rugogaster* ..................................A..C.........G....................................................

*Liolope* ..................................G............A....................................................

*Notocotylu*s .................................C.............G....................................................

*Echinostoma* ....................................................................................................

*Dicrogaster* .................................AG.................................................................

Consensus ATCCGAAGTAATGGTTAAGAGGGACAGACGGGGGCATTTGTATGGCGGTGTTAGAGGTGAAATTCTTGGATCGCCGCCAGACAAACTACAGCGAAAGCAT

*Transversotrema* T.......................................................................AT..............A...........

*Robinia* ..........................A.....................C................A.......T..........................

*Trichobilharzia* ..................................................................G.....AT..........................

*Rugogaster* .....................A..........................C...................................................

*Liolope* .............................................T......................................................

*Notocotylus* ....................................................................................................

*Echinostoma* ....................................................................................................

*Dicrogaster* ....................................................................................................

Nem18SlongR

<TTCATTAATCAAGAACGAAAGTC

Consensus TTGCCAAGGATG-TTTTCATTGATCTGGAGCGAAAGTC

*Transversotrema* ............T............AA......C....

*Robinia* ............-.........................

*Trichobilharzia* ........A...-............A............

*Rugogaster* ........A...-.............T...........

*Liolope* ........A...-............A............

*Notocotylus* ............-........................T

*Echinostoma* ............-.............T...........

*Dicrogaster* ............-.........................
