# Supplementary material for: A Megafauna’s Microfauna: Gastrointestinal Parasites of New Zealand’s Extinct Moa (Aves: Dinornithiformes)
Source: PLoS One. 2013 Feb 25;8(2):e57315. doi: 10.1371/journal.pone.0057315 (PMC3581471; doi:10.1371/journal.pone.0057315)
Supplement: Figure S4 — Alignment of Apicomplexa 18S sequences for designing the Nem18SlongF and Nem18SlongR primers. (DOC) [file pone.0057315.s004.doc]

**Nem18SlongF**

**CAGGGCAAGTCTGGTGCCAGCAGC>**

**APICOMPLEXA**

Consensus GAGGGCAAGTCTGGTGCCAGCAGCCGCGGTAATTCCAGCTCCAATAGYGTATATTAGAGTTGTTGCAGTTAAAAAGCTCGTAGTTGGATTTC--------

*Plasmodium* ...............................................C........A.A...............C...........A.....AAAGAATC

*Cryptosporidium* ...............................................C........A...................................--------

*Calyptospora* .....A.........................................C.......T....................................--------

*Toxoplasma* ...............................................C........A...................................--------

*Lankesterella* ...............................................T............................................--------

*Eimeria* ...............................................T............................................--------

*Isospora* ...............................................T............................................--------

*Cyclospora* ...............................................T............................................--------

Consensus --TG---------TCGTGGTCA-----TCCKGTACCRCCCGTATGGGTGTGCACTTG-KTTTGMYCTCG---GCATTCTTCCGGTAG-------------

*Plasmodium* GA.A---------.TT.AAG..ACGCT..TA.CTTAAT..AC..AAC..-AT....CGTA.C.ACT.T.TGC.....T.G.TAT..T-------------

*Cryptosporidium* --..---------.T..A--T.-----ATTTA..ATATTACC.A..-----T.A..A-T.A.ATTA..A---A...C.....TA.T--------------

*Calyptospora* --..TGTCGATTC...C..ATG-----C.AT...ATATAT-..CT.....TG.....CTG.GAGT.GGT---.....T.CT.A.CT.TGTGATATGTTGT

*Toxoplasma* --..---------CT.GAAG..-----G..A..-..G...TC.G.............-G-.GAATTCTA---....C....T..ATT-------------

*Lankesterella* --..---------C....A..G-----...G.C...A...C..G.....-ATGT.A.-GA.G.ATTGT.---....AA....A....-------------

*Eimeria* --..---------........T-----...T..G.TG..........C......G..-G....CC....---A.T............-------------

*Isospora* --..---------.........-----...G.....G............C....C..-G....CC....---..T............-------------

*Cyclospora* --..---------.........-----.G.G.CCG.G......-........G.C..-TG...CC.G..---..T............-------------

Consensus ---------CN--TTCCGCGCTTCATTG----------CGTGN--GAKG------------GTGTT---------CTGGAACTT----TTACTTTGAGAA

*Plasmodium* -------------G.GTT.TT..A...AAAATGATTCCTT.TAAG..CT------------T.C..TGCTTTGGCT.....G.CCTTG..........T.

*Cryptosporidium* -------------------------------------------------------------A.A..CTAAATATATA....A..----............

*Calyptospora* GTGATAATT.TGAC.GT.GAT..A...A----------TT..CAA..G.AACACACATAGC..A..ATATACCCG...AG....----............

*Toxoplasma* ---------TC---...A.A........----------T...---..GT------------T.T..---------.CA.G....----............

*Lankesterella* ---------.TCG..G.C.....A....----------....GCAAGG.------------.....---------.........----............

*Eimeria* ---------.C--.C.T........C..----------....G--.CT.------------.....---------.........----............

*Isospora* ---------.C--............C..----------....T--.TT.------------.....---------.C.......----............

*Cyclospora* -----------CC............C..----------....C--.TT.------------.....---------.C.......----............

Consensus AAATAGAGTGTTTCAAGCAGGC--TTGTCGC--------CYTGAATACTGCAGCATGGAATAATAAGATAGGAC---------YTTGGTTCTATTTTGTT

*Plasmodium* ..T.........CA.....AA.AGA.A.A..ATTGCGCGTT........A.............C..A..T.A..AAGTCAGAAT..T.....T....C..

*Cryptosporidium* ..T.......C..A........---AACT..--------.T........C.................TA.....---------T..T.-...T.C..A..

*Calyptospora* ..............TG......TT..T.T..--------.T.........................TT......---------T................

*Toxoplasma* ..T................-..--.......--------.T................................T---------T.C..CC..........

*Lankesterella* ......................--.......--------.C...........C.....................---------CC...............

*Eimeria* ......................--.......--------.C.................................---------C.C..............

*Isospora* ......................--.......--------.C........T........................---------C.C..............

*Cyclospora* ......................--.......--------.C........T........................---------C................

Consensus GGTTTCTAGGACCAARGTAATGATTAATAGGGACAG-TTGGGGGCA----TTCGTATTTAACTGT--CAGAGGTGAAAT-TCTTAGATT-TGTTAAAGAC

*Plasmodium*  AT...----.G.TT.-..T.C..........AGT..C.........----..T.....C.GA...--............-.........-.TC.GG....

*Cryptosporidium* ..-.........A..A......G.............-.........----............A.C--............-.........-..........

*Calyptospora* ............T.GA...G................-.........----.........T..G..--............-.........-C..A......

*Toxoplasma* ............TG.A..................G.-.........----...............--............-.........-..........

*Lankesterella* ..C....G.....G.C....................-...C.C..GCGCC.....C.........CA............C.....C...C..........

*Eimeria* ...............G....................-.........----...............--............-.........-..........

*Isospora* ...............G....................-.........----...............--............-.........-..........

*Cyclospora* .............G.G....................-.........----...............--............-.........-..........

Nem18SlongR

<TTCATTAATCAAGAACGAAAGTC

Consensus GAACTACTGCGAAAGCATTTGCCAA-GGATGTTTTCATTAATCAAGAACGACAGTA

*Plasmodiu*m A...A..................T.-AA..AC..C................A...T

*Cryptosporidium* .........................-.........................A...T

*Calyptospora* .........................-..............................

*Toxoplasma* .........................-A........................A...T

*Lankesterella* ...-.....T...............G..............................

*Eimeria* .........................-..............................

*Isospora* .........................-..............................

*Cyclospora* .........................-..............................
