# Supplementary material for: A Megafauna’s Microfauna: Gastrointestinal Parasites of New Zealand’s Extinct Moa (Aves: Dinornithiformes)
Source: PLoS One. 2013 Feb 25;8(2):e57315. doi: 10.1371/journal.pone.0057315 (PMC3581471; doi:10.1371/journal.pone.0057315)
Supplement: Figure S5 — Alignment of clone sequences obtained from moa coprolites using Nem18SF and Nem18SR primers. (DOC) [file pone.0057315.s005.doc]

**1 50 100**

Consensus: TTTWRCCTGCTAAATAGTWTG--SCKRTCMTY---TGTGNNNNNNNNNNNNNNNNNNNNNNNNNNNNNNNNNNNNNNNAGNGGANTCGTTGTYCGWCGGGTGCGGCGCAGGTAATT

**Sequence 1 (*Cryptosporidium*)**

10198_Megalapteryx_Dart: C..AA.............CAC---------------------------------------------------------..GAA.T.....T.CT.GTA.-----------------

10198_Megalapteryx_Dart: C..AA.............CAC---------------------------------------------------------..GAA.T.....T.CT.GTA.-----------------

10198_Megalapteryx_Dart: C..AA.............CAC---------------------------------------------------------..GAA.T.....T.CT.GTA.-----------------

10198_Megalapteryx_Dart: C..AA.............CAC---------------------------------------------------------..GAA.T.....T.CT.GTA.-----------------

10198_Megalapteryx_Dart: C..AA.............CAC---------------------------------------------------------..GAA.T.....T.CT.GTA.-----------------

10198_Megalapteryx_Dart: C..AA.............CAC---------------------------------------------------------..GAA.T.....T.CT.GTA.-----------------

**Sequence 2 group (Eimeriorina sp. 1)**

10500_Megalapteryx_Dart: C..AG............GA.C--GGGAA.T.A---...--------------------------------------------------..C.G.AT.-------------------

10500_Megalapteryx_Dart: C..AG............GA.C--GGGAA.T.A---...--------------------------------------------------..C.G.AT.-------------------

10500_Megalapteryx_Dart: C..AG............GA.C--GGGAA.T.A---...--------------------------------------------------..C.G.AT.-------------------

10198_Megalapteryx_Dart: C..AG............GA.C--GGGAA.T.A---...--------------------------------------------------..C.G.AT.-------------------

10198_Megalapteryx_Dart: C..AG............GA.C--GGGAA.T.A---...--------------------------------------------------..C.G.AT.-------------------

10504_Megalapteryx_Dart: C..AG............GA.C--GGGAA.T.T--A...--------------------------------------------------..C.G.AT.-------------------

10198_Megalapteryx_Dart: C..AG............GA.C--GGGAA.T.T--A...--------------------------------------------------..C.G.AT.-------------------

10146_Megalapteryx_Euphrates: C..AG............GA.C--GGGAA.T.T--A...--------------------------------------------------..C.G.AT.-------------------

10146_Megalapteryx_Euphrates: C..AG............GA.C--GGGAA.T.T--A...--------------------------------------------------..C.G.AT.-------------------

10146_Megalapteryx_Euphrates: C..AG............GA.C--GGGAA.T.T--A...--------------------------------------------------..C.G.AT.-------------------

10146_Megalapteryx_Euphrates: C..AG............GA.C--GGGAA.T.T--A...--------------------------------------------------..C.G.AT.-------------------

10197_Megalapteryx_Dart: C..AG............GA.C--GGGAA.T.T---...--------------------------------------------------..C.G.AT.-------------------

10146_Megalapteryx_Euphrates: C..AG............GA.C--GGGAA.T.T---...--------------------------------------------------..C.G.AT.-------------------

10146_Megalapteryx_Euphrates: C..AG............GA.C--GGGAA.T.T---...--------------------------------------------------..C.G.AT.-------------------

10146_Megalapteryx_Euphrates: C..AG............GA.C--GGGAA.T.T---...--------------------------------------------------..C.G.AT.-------------------

10146_Megalapteryx_Euphrates: C..AG............GA.C--GGGAA.T.C--A...--------------------------------------------------..C.G.AT.-------------------

10146_Megalapteryx_Euphrates: C..AG............GA.C--GGGAA.T.C--A...--------------------------------------------------..C.G.AT.-------------------

10146_Megalapteryx_Euphrates: C..AG............GA.C--GGGAA.T.C--A...--------------------------------------------------..C.G.AT.-------------------

10146_Megalapteryx_Euphrates: C..AG............GA.C--GGGAA.T.C--A...--------------------------------------------------..C.G.AT.-------------------

10146_Megalapteryx_Euphrates: C..AG............GA.C--GGGAA.T.C--A...--------------------------------------------------..C.G.AT.-------------------

10146_Megalapteryx_Euphrates: C..AG............GA.C--GGGAA.T.C--A...--------------------------------------------------..C.G.AT.-------------------

10146_Megalapteryx_Euphrates: C..AG............GA.C--GGGAA.T.C--A...--------------------------------------------------..C.G.AT.-------------------

10146_Megalapteryx_Euphrates: C..AG............GA.C--GGGAA.T.C--A...--------------------------------------------------..C.G.AT.-------------------

10198_Megalapteryx_Dart: C..AG............GA.C--GGGAAAT.A---G..--------------------------------------------------..C.G.AT.-------------------

**Sequence group 4 (Heterakoidea)**

10503_Dinornis_Dart: .C.GA...A.........G.C--TGGA.AA.T--A....CAGACG-----------------------------------------------------------------------

10503_Dinornis_Dart: .C.GA...A.........G.C--TGGA.AA.T--A....CAGACG-----------------------------------------------------------------------

10503_Dinornis_Dart: .C.GA...A.........G.C--TGGA.AA.T--A....CAGACG-----------------------------------------------------------------------

10501_Anomalopteryx_Dart: .C.GA...A.........G.C--TGGA.AA.T--A....CAGACG-----------------------------------------------------------------------

10198_Megalapteryx_Dart: .C.GA...A.........G.C--TGGA.AA.T--A....CAGACG-----------------------------------------------------------------------

10142_Megalapteryx_Euphrates: .C.GA...A.........G.C--TGGA.AA.T--A....CAGACG-----------------------------------------------------------------------

10501_Anomalopteryx_Dart: .C.GA...A.........G.C--TGGA.AACT--A....CAGACG-----------------------------------------------------------------------

10503_Dinornis_Dart: .C.GA...A.........G.C--TGGA.AA.T--A....CAGATG-----------------------------------------------------------------------

10503_Dinornis_Dart: .C.GA...A.........G.C--TGGA.AA.T--A....CAGATG-----------------------------------------------------------------------

10503_Dinornis_Dart: .C.GA...A.........G.C--TGGA.AA.T--A....CAGATG-----------------------------------------------------------------------

10503_Dinornis_Dart: .C.GA...A.........G.C--TGGA.AA.T--A....CAGATG-----------------------------------------------------------------------

10503_Dinornis_Dart: .C.GA...A.........G.C--TGGA.AA.T--A....CAGATG-----------------------------------------------------------------------

10503_Dinornis_Dart: .C.GA...A.........G.C--TGGA.AA.T--A....CAGATG-----------------------------------------------------------------------

10503_Dinornis_Dart: .C.GA...A.........G.C--TGGA.AA.T--A....CAGATG-----------------------------------------------------------------------

10503_Dinornis_Dart: .C.GA...A.........G.C--TGGA.AA.T--A....CAGATG-----------------------------------------------------------------------

10503_Dinornis_Dart: .C.GA...A.........G.C--TGAA.AA.T--A....CAGATG-----------------------------------------------------------------------

10503_Dinornis_Dart: .C.GA...A.........G.C--TGAA.AA.T--A....CAGATG-----------------------------------------------------------------------

10503_Dinornis_Dart: .C.GA...A.........G.C--TGAA.AA.T--A....CAGATG-----------------------------------------------------------------------

10503_Dinornis_Dart: .C.GA...A.........G.C--TGAA.AA.T--A....CAGACG-----------------------------------------------------------------------

10503_Dinornis_Dart: .C.GA...A.........G.C--TGAA.AA.T--A....CAGACG-----------------------------------------------------------------------

10503_Dinornis_Dart: .C.GA...A.........G.C--TGAA.AA.T--A....CAGACG-----------------------------------------------------------------------

10503_Dinornis_Dart: .C.GA...A.........G.C--TGAA.AA.T--A....CAGACG-----------------------------------------------------------------------

10503_Dinornis_Dart: .C.GA...A.........G.C--TGAA.AA.T--A....CAGACG-----------------------------------------------------------------------

10503_Dinornis_Dart: .C.GA...A.........G.C--TGAA.AA.T--A....CAGACG-----------------------------------------------------------------------

10503_Dinornis_Dart: .C.GA...A.........G.C--TGAA.AA.T--A....CAGACG-----------------------------------------------------------------------

10503_Dinornis_Dart: .C.GA...A.........G.C--TGAA.AA.T--A....CAGACG-----------------------------------------------------------------------

10503_Dinornis_Dart: .C.GA...A.........G.C--TGAA.AA.T--A....CAGACG-----------------------------------------------------------------------

10192_Pachyornis_Dart: .C.GA...A.........G.C--TGAA.AA.T--A....CAGACG-----------------------------------------------------------------------

10190_Dinornis_Dart: .C.GA...A.........G.C--TGAA.AA.T--A....CAGACG-----------------------------------------------------------------------

10189_Dinornis_Dart: .C.GA...A.........G.C--TGAA.AA.T--A....CAGACG-----------------------------------------------------------------------

10189_Dinornis_Dart: .C.GA...A.........G.C--TGAA.AA.T--A....CAGACG-----------------------------------------------------------------------

10503_Dinornis_Dart: .C.GA...A.........G.C--TGAA.AA.T--A....CAGACG-----------------------------------------------------------------------

10501_Anomalopteryx_Dart: .C.GA...A.........G.C--TGAA.AA.TTT--...CAGACG-----------------------------------------------------------------------

10501_Anomalopteryx_Dart: .C.GA...A.........G.C--TGAA.AA.TTT----.CAGACG-----------------------------------------------------------------------

**Coprolite sequence 5 (Trichostrongylidae)**

10142_Megalapteryx_Euphrates: .C.AG.............GGC--TGGA.TT.T---------------------------------------------A..TCC.G..T----------------------------

**Coprolite sequence 6a group (Echinostomida: Notocotylidae?)**

10504_Megalapteryx_Dart: ...TG.............A..--C.TG..C.C---....CTCGTTCAGGTCACGATATAAGCTGCCTCCTTGTGGGGT..C...G.......C..A....................

10504_Megalapteryx_Dart: ...TG.............A..--C.TG..C.C---....CTCGTTCAGGTCACGATATAAGCTGCCTCCTTGTGGGGT..C...G.......C..A....................

10504_Megalapteryx_Dart: ...TG.............A..--C.TG..C.C---....CTCGTTCAGGTCACGATATAAGCTGCCTCCTTGTGGGGT..C...G.......C..A....................

10504_Megalapteryx_Dart: ...TG.............A..--C.TG..C.C---....CTCGTTCAGGTCACGATATAAGCTGCCTCCTTGTGGGGT..C...G.......C..A....................

10504_Megalapteryx_Dart: ...TG.............A..--C.TG..C.C---....CTCGTTCAGGTCACGATATAAGCTGCCTCCTTGTGGGGT..C...G.......C..A....................

10504_Megalapteryx_Dart: ...TG.............A..--C.TG..C.C---....CTCGTTCAGGTCACGATATAAGCTGCCTCCTTGTGGGGT..C...G.......C..A....................

10504_Megalapteryx_Dart: ...TG.............A..--C.TG..C.C---....CTCGTTCAGGTCACGATATAAGCTGCCTCCTTGTGGGGT..C...G.......C..A....................

10504_Megalapteryx_Dart: ...TG.............A..--C.TG..C.C---....CTCGTTCAGGTCACGATATAAGCTGCCTCCTTGTGGGGT..C...G.......C..A....................

10504_Megalapteryx_Dart: ...TG.............A..--C.TG..C.C---....CTCGTTCAGGTCACGATATAAGCTGCCTCCTTGTGGGGT..C...G.......C..A....................

10504_Megalapteryx_Dart: ...TG.............A..--C.TG..C.C---....CTCGTTCAGGTCACGATATAAGCTGCCTCCTTGTGGGGT..C...G.......C..A....................

10504_Megalapteryx_Dart: ...TG.............A..--C.TG..C.C---....CTCGTTCAGGTCACGATATAAGCTGCCTCCTTGTGGGGT..C...G.......C..A....................

10504_Megalapteryx_Dart: ...TG.............A..--C.TG..C.C---....CTCGTTCAGGTCACGATATAAGCTGCCTCCTTGTGGGGT..C...G.......C..A....................

10504_Megalapteryx_Dart: ...TG.............A..--C.TG..C.C---....CTCGTTCAGGTCACGATATAAGCTGCCTCCTTGTGGGGT..C...G.......C..A....................

10504_Megalapteryx_Dart: ...TG.............A..--C.TG..C.C---....CTCGTTCAGGTCACGATATAAGCTGCCTCCTTGTGGGGT..C...G.......C..A....................

10500_Megalapteryx_Dart: ...TG.............A..--C.TG..C.C---....CTCGTTCAGGTCACGATATAAGCTGCCTCCTTGTGGGGT..C...G.......C..A....................

10500_Megalapteryx_Dart: ...TG.............A..--C.TG..C.C---....CTCGTTCAGGTCACGATATAAGCTGCCTCCTTGTGGGGT..C...G.......C..A....................

10500_Megalapteryx_Dart: ...TG.............A..--C.TG..C.C---....CTCGTTCAGGTCACGATATAAGCTGCCTCCTTGTGGGGT..C...G.......C..A....................

10500_Megalapteryx_Dart: ...TG.............A..--C.TG..C.C---....CTCGTTCAGGTCACGATATAAGCTGCCTCCTTGTGGGGT..C...G.......C..A....................

10500_Megalapteryx_Dart: ...TG.............A..--C.TG..C.C---....CTCGTTCAGGTCACGATATAAGCTGCCTCCTTGTGGGGT..C...G.......C..A....................

10500_Megalapteryx_Dart: ...TG.............A..--C.TG..C.C---....CTCGTTCAGGTCACGATATAAGCTGCCTCCTTGTGGGGT..C...G.......C..A....................

10500_Megalapteryx_Dart: ...TG.............A..--C.TG..C.C---....CTCGTTCAGGTCACGATATAAGCTGCCTCCTTGTGGGGT..C...G.......C..A....................

10500_Megalapteryx_Dart: ...TG.............A..--C.TG..C.C---....CTCGTTCAGGTCACGATATAAGCTGCCTCCTTGTGGGGT..C...G.......C..A....................

10500_Megalapteryx_Dart: ...TG.............A..--C.TG..C.C---....CTCGTTCAGGTCACGATATAAGCTGCCTCCTTGTGGGGT..C...G.......C..A....................

10500_Megalapteryx_Dart: ...TG.............A..--C.TG..C.C---....CTCGTTCAGGTCACGATATAAGCTGCCTCCTTGTGGGGT..C...G.......C..A....................

10500_Megalapteryx_Dart: ...TG.............A..--C.TG..C.C---....CTCGTTCAGGTCACGATATAAGCTGCCTCCTTGTGGGGT..C...G.......C..A....................

10500_Megalapteryx_Dart: ...TG.............A..--C.TG..C.C---....CTCGTTCAGGTCACGATATAAGCTGCCTCCTTGTGGGGT..C...G.......C..A....................

10500_Megalapteryx_Dart: ...TG.............A..--C.TG..C.C---....CTCGTTCAGGTCACGATATAAGCTGCCTCCTTGTGGGGT..C...G.......C..A....................

10500_Megalapteryx_Dart: ...TG.............A..--C.TG..C.C---....CTCGTTCAGGTCACGATATAAGCTGCCTCCTTGTGGGGT..C...G.......C..A....................

10500_Megalapteryx_Dart: ...TG.............A..--C.TG..C.C---....CTCGTTCAGGTCACGATATAAGCTGCCTCCTTGTGGGGT..C...G.......C..A....................

10500_Megalapteryx_Dart: ...TG.............A..--C.TG..C.C---....CTCGTTCAGGTCACGATATAAGCTGCCTCCTTGTGGGGT..C...G.......C..A....................

10500_Megalapteryx_Dart: ...TG.............A..--C.TG..C.C---....CTCGTTCAGGTCACGATATAAGCTGCCTCCTTGTGGGGT..C...G.......C..A....................

10500_Megalapteryx_Dart: ...TG.............A..--C.TG..C.C---....CTCGTTCAGGTCACGATATAAGCTGCCTCCTTGTGGGGT..C...G.......C..A....................

10198_Megalapteryx_Dart: ...TG.............A..--C.TG..C.C---....CTCGTTCAGGTCACGATATAAGCTGCCTCCTTGTGGGGT..C...G.......C..A....................

10198_Megalapteryx_Dart: ...TG.............A..--C.TG..C.C---....CTCGTTCAGGTCACGATATAAGCTGCCTCCTTGTGGGGT..C...G.......C..A....................

10198_Megalapteryx_Dart: ...TG.............A..--C.TG..C.C---....CTCGTTCAGGTCACGATATAAGCTGCCTCCTTGTGGGGT..C...G.......C..A....................

10198_Megalapteryx_Dart: ...TG.............A..--C.TG..C.C---....CTCGTTCAGGTCACGATATAAGCTGCCTCCTTGTGGGGT..C...G.......C..A....................

10198_Megalapteryx_Dart: ...TG.............A..--C.TG..C.C---....CTCGTTCAGGTCACGATATAAGCTGCCTCCTTGTGGGGT..C...G.......C..A....................

10198_Megalapteryx_Dart: ...TG.............A..--C.TG..C.C---....CTCGTTCAGGTCACGATATAAGCTGCCTCCTTGTGGGGT..C...G.......C..A....................

10198_Megalapteryx_Dart: ...TG.............A..--C.TG..C.C---....CTCGTTCAGGTCACGATATAAGCTGCCTCCTTGTGGGGT..C...G.......C..A....................

10198_Megalapteryx_Dart: ...TG.............A..--C.TG..C.C---....CTCGTTCAGGTCACGATATAAGCTGCCTCCTTGTGGGGT..C...G.......C..A....................

10197_Megalapteryx_Dart: ...TG.............A..--C.TG..C.C---....CTCGTTCAGGTCACGATATAAGCTGCCTCCTTGTGGGGT..C...G.......C..A....................

10197_Megalapteryx_Dart: ...TG.............A..--C.TG..C.C---....CTCGTTCAGGTCACGATATAAGCTGCCTCCTTGTGGGGT..C...G.......C..A....................

10197_Megalapteryx_Dart: ...TG.............A..--C.TG..C.C---....CTCGTTCAGGTCACGATATAAGCTGCCTCCTTGTGGGGT..C...G.......C..A....................

10197_Megalapteryx_Dart: ...TG.............A..--C.TG..C.C---....CTCGTTCAGGTCACGATATAAGCTGCCTCCTTGTGGGGT..C...G.......C..A....................

10197_Megalapteryx_Dart: ...TG.............A..--C.TG..C.C---....CTCGTTCAGGTCACGATATAAGCTGCCTCCTTGTGGGGT..C...G.......C..A....................

10197_Megalapteryx_Dart: ...TG.............A..--C.TG..C.C---....CTCGTTCAGGTCACGATATAAGCTGCCTCCTTGTGGGGT..C...G.......C..A....................

10197_Megalapteryx_Dart: ...TG.............A..--C.TG..C.C---....CTCGTTCAGGTCACGATATAAGCTGCCTCCTTGTGGGGT..C...G.......C..A....................

10197_Megalapteryx_Dart: ...TG.............A..--C.TG..C.C---....CTCGTTCAGGTCACGATATAAGCTGCCTCCTTGTGGGGT..C...G.......C..A....................

10197_Megalapteryx_Dart: ...TG.............A..--C.TG..C.C---....CTCGTTCAGGTCACGATATAAGCTGCCTCCTTGTGGGGT..C...G.......C..A....................

10197_Megalapteryx_Dart: ...TG.............A..--C.TG..C.C---....CTCGTTCAGGTCACGATATAAGCTGCCTCCTTGTGGGGT..C...G.......C..A....................

10197_Megalapteryx_Dart: ...TG.............A..--C.TG..C.C---....CTCGTTCAGGTCACGATATAAGCTGCCTCCTTGTGGGGT..C...G.......C..A....................

10197_Megalapteryx_Dart: ...TG.............A..--C.TG..C.C---....CTCGTTCAGGTCACGATATAAGCTGCCTCCTTGTGGGGT..C...G.......C..A....................

10197_Megalapteryx_Dart: ...TG.............A..--C.TG..C.C---....CTCGTTCAGGTCACGATATAAGCTGCCTCCTTGTGGGGT..C...G.......C..A....................

10197_Megalapteryx_Dart: ...TG.............A..--C.TG..C.C---....CTCGTTCAGGTCACGATATAAGCTGCCTCCTTGTGGGGT..C...G.......C..A....................

10197_Megalapteryx_Dart: ...TG.............A..--C.TG..C.C---....CTCGTTCAGGTCACGATATAAGCTGCCTCCTTGTGGGGT..C...G.......C..A....................

10197_Megalapteryx_Dart: ...TG.............A..--C.TG..C.C---....CTCGTTCAGGTCACGATATAAGCTGCCTCCTTGTGGGGT..C...G.......C..A....................

10197_Megalapteryx_Dart: ...TG.............A..--C.TG..C.C---....CTCGTTCAGGTCACGATATAAGCTGCCTCCTTGTGGGGT..C...G.......C..A....................

10197_Megalapteryx_Dart: ...TG.............A..--C.TG..C.C---....CTCGTTCAGGTCACGATATAAGCTGCCTCCTTGTGGGGT..C...G.......C..A....................

10197_Megalapteryx_Dart: ...TG.............A..--C.TG..C.C---....CTCGTTCAGGTCACGATATAAGCTGCCTCCTTGTGGGGT..C...G.......C..A....................

10197_Megalapteryx_Dart: ...TG.............A..--C.TG..C.C---....CTCGTTCAGGTCACGATATAAGCTGCCTCCTTGTGGGGT..C...G.......C..A....................

10197_Megalapteryx_Dart: ...TG.............A..--C.TG..C.C---....CTCGTTCAGGTCACGATATAAGCTGCCTCCTTGTGGGGT..C...G.......C..A....................

10173_Megalapteryx_Euphrates: ...TG.............A..--C.TG..C.C---....CTCGTTCAGGTCACGATATAAGCTGCCTCCTTGTGGGGT..C...G.......C..A....................

10173_Megalapteryx_Euphrates: ...TG.............A..--C.TG..C.C---....CTCGTTCAGGTCACGATATAAGCTGCCTCCTTGTGGGGT..C...G.......C..A....................

10152_Megalapteryx_Euphrates: ...TG.............A..--C.TG..C.C---....CTCGTTCAGGTCACGATATAAGCTGCCTCCTTGTGGGGT..C...G.......C..A....................

10152_Megalapteryx_Euphrates: ...TG.............A..--C.TG..C.C---....CTCGTTCAGGTCACGATATAAGCTGCCTCCTTGTGGGGT..C...G.......C..A....................

10152_Megalapteryx_Euphrates: ...TG.............A..--C.TG..C.C---....CTCGTTCAGGTCACGATATAAGCTGCCTCCTTGTGGGGT..C...G.......C..A....................

10504_Megalapteryx_Dart: ...TG.............A..--C.TG..C.C---....CTCGTTCAGGTCACGATATAAGCTGCCTCCTTGTGGGGT..C...G....C..C..A....................

10504_Megalapteryx_Dart: ...TG.............A..--C.TG..C.C---....CTCGTTCAGGTCACGATATAGGCTGCCTCCTTGTGGGGT..C...G.......C..A....................

10500_Megalapteryx_Dart: ...TG.............A..--C.TG..C.C---....CTCGTTCAGGTCACGATATAAGCTGCCTCCTTGTGGGGT..C...G.......C..A....C...............

10500_Megalapteryx_Dart: ...TG.............A..--C.TG..C.C---....CTCGTTCAGGTCACGATATAAGCTGCCTCCTTGTGGGGT..C...G.......C..A....C...............

10500_Megalapteryx_Dart: ...TG.............A..--C.TG..C.C---....CTCGTTCAGGTCACGATATAAGCTGCCTCCTTGTGGGAT..C...G.......C..A....................

10152_Megalapteryx_Euphrates: ...TG.............A..--C.TG..C.C---....CTCGTTCAGGTCACGATGTATGCTGCCTCCTTGTGGGGT..C...G.......C..A....................

10142_Megalapteryx_Euphrates: ...TG.............A..--C.TG..C.C---....CTCGTTCAGGGCACGATGTATGCTGCCTCCTTGTGGGGT..C...G.......C..A....................

**Coprolite sequence 6b group (Echinostomida: Notocotylidae?)**

10146_Megalapteryx_Euphrates: ...TG....T........A..--C.TG..C.C---....CTCGTTCAGGGCACGATGTATGCTGCCTCCTTGTGGGAT..T...G.......T..A..A.................

10146_Megalapteryx_Euphrates: ...TG.............A..--C.TG..C.C---....CTCGTTAAGGGCACGATGTATGCTGCCTCCTTGTGGGAT..T...G.......T..A..A.................

10173_Megalapteryx_Euphrates: ...TG.............A..--C.TG..C.C---....CTCGTTCAGGGCACGATGTATGCTGCCTCCTTGTGGGGT..C...G.......T..A..A.................

10173_Megalapteryx_Euphrates: ...TG.............A..--C.TG..C.C---....CTCGTTCAGGGCACGATGTATGCTGCCTCCTTGTGGGGT..C...G.......T..A..A.................

10173_Megalapteryx_Euphrates: ...TG.............A..--C.TG..C.C---....CTCGTTCAGGGCACGATGTATGCTGCCTCCTTGTGGGGT..C...G.......T..A..A.................

10173_Megalapteryx_Euphrates: ...TG.............A..--C.TG..C.C---....CTCGTTCAGGGCACGATGTATGCTGCCTCCTTGTGGGGT..C...G.......T..A..A.................

10152_Megalapteryx_Euphrates: ...TG.............A..--C.TG..C.C---....CTCGTTCAGGGCACGATGTATGCTGCCTCCTTGTGGGGT..C...G.......T..A..A.................

10146_Megalapteryx_Euphrates: ...TG.............A..--C.TG..C.C---....CTCGTTCAGGGCACGATGTATGCTGCCTCCTTGTGGGGT..C...G.......T..A..A.................

10146_Megalapteryx_Euphrates: ...TG.............A..--C.TG..C.C---....CTCGTTCAGGGCACGATGTATGCTGCCTCCTTGTGGGGT..C...G.......T..A..A.................

10146_Megalapteryx_Euphrates: ...TG.............A..--C.TG..C.C---....CTCGTTCAGGGCACGATGTATGCTGCCTCCTTGTGGGGT..C...G.......T..A..A.................

10142_Megalapteryx_Euphrates: ...TG.............A..--C.TG..C.C---....CTCGTTCAGGGCACGATGTATGCTGCCTCCTTGTGGGGT..C...G.......T..A..A.................

10142_Megalapteryx_Euphrates: ...TG.............A..--C.TG..C.C---....CTCGTTCAGGGCACGATGTATGCTGCCTCCTTGTGGGGT..C...G.......T..A..A.................

10142_Megalapteryx_Euphrates: ...TG.............A..--C.TG..C.C---....CTCGTTCAGGGCACGATGTATGCTGCCTCCTTGTGGGGT..C...G.......T..A..A.................

10142_Megalapteryx_Euphrates: ...TG.............A..--C.TG..C.C---....CTCGTTCAGGGCACGATGTATGCTGCCTCCTTGTGGGGT..C...G.......T..A..A.................

10142_Megalapteryx_Euphrates: ...TG.............A..--C.TG..C.C---....CTCGTTCAGGGCACGATGTATGCTGCCTCCTTGTGGGGT..C...G.......T..A..A.................

10142_Megalapteryx_Euphrates: ...TG.............A..--C.TG..C.C---....CTCGTTCAGGGCACGATGTATGCTGCCTCCTTGTGGGGT..C...G.......T..A..A.................

10142_Megalapteryx_Euphrates: ...TG.............A..--C.TG..C.C---....CTCGTTCAGGGCACGATGTATGCTGCCTCCTTGTGGGGT..C...G.......T..A..A.................

10142_Megalapteryx_Euphrates: ...TG.............A..--C.TG..C.C---....CTCGTTCAGGGCACGATGTATGCTGCCTCCTTGTGGGGT..C...G.......T..A..A.................

10142_Megalapteryx_Euphrates: ...TG.............A..--C.TG..C.C---....CTCGTTCAGGGCACGATGTATGCTGCCTCCTTGTGGGGT..C...G.......T..A..A.................

10142_Megalapteryx_Euphrates: ...TG.............A..--C.TG..C.C---....CTCGTTCAGGGCACGATGTATGCTGCCTCCTTGTGGGGT..C...G.......T..A..A.................

10142_Megalapteryx_Euphrates: ...TG.............A..--C.TG..C.C---....CTCGTTCAGGGCACGATGTATGCTGCCTCCTTGTGGGGT..C...G.......T..A..A.................

10142_Megalapteryx_Euphrates: ...TG.............A..--C.TG..C.C---....CTCGTTCAGGGCACGATGTATGCTGCCTCCTTGTGGGGT..C...G.......T..A..A.................

10142_Megalapteryx_Euphrates: ...TG.............A..--C.TG..C.C---....CTCGTTCAGGGCACGATGTATGCTGCCTCCTTGTGGGGT..C...G.......T..A..A.................

10142_Megalapteryx_Euphrates: ...TG.............A..--C.TG..C.C---....CTCGTTCAGGGCACGATGTATGCTGCCTCCTTGTGGGGT..C...G.......T..A..A.................

10142_Megalapteryx_Euphrates: ...TG.............A..--C.TG..C.C---....CTCGTTCAGGGCACGATGTATGCTGCCTCCTTGTGGGGT..C...G.......T..A..A.................

10142_Megalapteryx_Euphrates: ...TG.............A..--C.TG..C.C---....CTCGTTCAGGGCACGATGTATGCTGCCTCCTTGTGGGGT..C...G.......T..A..A.................

10173_Megalapteryx_Euphrates: ...TG.............A..--C.TG..C.C---....CTCGTTTAGGGCACGATGTATGCTGCCTCCTTGTGGGGT..C...G.......T..A..A.................

10146_Megalapteryx_Euphrates: ...TG....T........A..--C.TG..C.C---....CTCGTTCAGGGCACGATGTATGCTGCCTCCTTGTGGGGT..C...G.......T..A..A.................

10146_Megalapteryx_Euphrates: ...TG.............A..--C.TG..C.C---....CTCGTTCAGGGCACGATGTATGCTGCCTCCTTGTGGGAT..C...G.......T..A..A.................

**Non-target (Moa)**

10197_Megalapteryx_Dart: .C.GG.A......C....TAC--G.GAC.CCC-----------------------------------------------.A.CGG...GC..C.A---------------------

**Non-target (Fish)**

10142_Megalapteryx_Euphrates: .CCGG.A......C....TAT--G.GGC.CCG------------------------------------------------A.CGG...GC..C.A---------------------

10197_Megalapteryx_Dart: .CCTC.A......C....TAC--G.GGC.CCT------------------------------------------------C.TGG...GC..TTCA--------------------

10197_Megalapteryx_Dart: .CCTC.A...........TAC--G.GGC.CCT------------------------------------------------C.TGG...GC..TTCA--------------------

10197_Megalapteryx_Dart: .CCTC.A...........TAC--G.GGC.CCT------------------------------------------------C.TGG...GC..TTCA--------------------

10197_Megalapteryx_Dart: .CCTC.A...........TAC--G.GGC.CCT------------------------------------------------C.TGG...GC..TTCA--------------------

10152_Megalapteryx_Euphrates: .CCTC.A...........TAC--G.GGC.CCT------------------------------------------------C.TGG...GC..TTCA--------------------

10142_Megalapteryx_Euphrates: .CCTC.A...........TAC--G.GGC.CCT------------------------------------------------C.TGG...GC..TTCA--------------------

10142_Megalapteryx_Euphrates: .CCTC.A...........TAC--G.GGC.CCT------------------------------------------------C.TGG...GC..TTCA--------------------

10173_Megalapteryx_Euphrates: C.CAG........C....TAC--A.GA-----------------------------------------------------A...TCTCC.TCGT.G.CA-----------------

**Non-target (Plant)**

10197_Megalapteryx_Dart: C..AG........C...CTAT----------------------------------------------------------.T...GGTA.CCCT.CA...CCA--------------

10201_Pachyornis_Dart: C..AA........C....TAC--C.---------------------------------------------------------------....GTTAATA.G.G.CTA---------

**Non-target (Ciliophora?)**

10201_Pachyornis_Dart: C..AA........C....TAC--C.TTATG.T-------------------------------------------AAT..G..GC.A-----------------------------

10201_Pachyornis_Dart: C..AA........C....TAC--C.TTATG.T-------------------------------------------AAT..G..GC.A-----------------------------

10201_Pachyornis_Dart: C..AA........C....TAC--C.TTATG.T-------------------------------------------AAT..G..GC.A-----------------------------

10164_Megalapteryx_Euphrates: C..AA........C....TAC--C.TTATG.T-------------------------------------------AAT..G..GC.A-----------------------------

**Non-target (Fungi)**

10501_Anomalopteryx_Dart: C..AA.............TA.--G.CAA.G.T------------------------------------------------------T....GT..T.A------------------

10501_Anomalopteryx_Dart: C..AA.............TA.--G.CAA.G.T------------------------------------------------------T....GT..T.A------------------

10501_Anomalopteryx_Dart: C..AA.............TA.--G.CAA.G.T------------------------------------------------------T....GT..T.A------------------

10501_Anomalopteryx_Dart: C..AA.............TA.--G.CAA.G.T------------------------------------------------------T....GT..T.A------------------

10501_Anomalopteryx_Dart: C..AA.............TA.--G.CAA.G.T------------------------------------------------------T....GT..T.A------------------

10501_Anomalopteryx_Dart: C..AA.............TA.--G.CAA.G.T------------------------------------------------------T....GT..T.A------------------

10501_Anomalopteryx_Dart: C..AA.............TA.--G.CAA.G.T------------------------------------------------------T....GT..T.A------------------

10501_Anomalopteryx_Dart: C..AA.............TA.--G.CAA.G.T------------------------------------------------------T....GT..T.A------------------

10501_Anomalopteryx_Dart: C..AA.............TA.--G.CAA.G.T------------------------------------------------------T....GT..T.A------------------

10501_Anomalopteryx_Dart: C..AA.............TA.--G.CAA.G.T------------------------------------------------------T....GT..T.A------------------

10501_Anomalopteryx_Dart: C..AA.............TA.--G.CAA.G.T------------------------------------------------------T....GT..T.A------------------

10501_Anomalopteryx_Dart: C..AA.............TA.--G.CAA.G.T------------------------------------------------------T....GT..T.A------------------

10201_Pachyornis_Dart: C..AA.............TA.--G.CAA.G.T------------------------------------------------------T....GT..T.A------------------

10201_Pachyornis_Dart: C..AA.............TA.--G.CAA.G.T------------------------------------------------------T....GT..T.A------------------

10201_Pachyornis_Dart: C..AA.............TA.--G.CAA.G.T------------------------------------------------------T....GT..T.A------------------

10201_Pachyornis_Dart: C..AA.............TA.--G.CAA.G.T------------------------------------------------------T....GT..T.A------------------

10164_Megalapteryx_Euphrates: C..AA.............TA.--G.CAA.G.T------------------------------------------------------T....GT..T.A------------------

10164_Megalapteryx_Euphrates: C..AA.............TA.--G.CAA.G.T------------------------------------------------------T....GT..T.A------------------

10164_Megalapteryx_Euphrates: C..AA.............TA.--G.CAA.G.T------------------------------------------------------T....GT..T.A------------------

10164_Megalapteryx_Euphrates: C..AA.............TA.--G.CAA.G.T------------------------------------------------------T....GT..T.A------------------

10164_Megalapteryx_Euphrates: C..AA.............TA.--G.CAA.G.T------------------------------------------------------T....GT..T.A------------------

10164_Megalapteryx_Euphrates: C..AA.............TA.--G.CAA.G.T------------------------------------------------------T....GT..T.A------------------

10164_Megalapteryx_Euphrates: C..AA.............TA.--G.CAA.G.T------------------------------------------------------T....GT..T.A------------------

10164_Megalapteryx_Euphrates: C..AA.............TA.--G.CAA.G.T------------------------------------------------------T....GT..T.A------------------

10164_Megalapteryx_Euphrates: C..AA.............TA.--G.CAA.G.T------------------------------------------------------T....GT..T.A------------------

10164_Megalapteryx_Euphrates: C..AA.............TA.--G.CAA.G.T------------------------------------------------------T....GT..T.A------------------

10501_Anomalopteryx_Dart: C..AA.............TA.--G.CAA.G.C------------------------------------------------------T....GT..T.A------------------

10501_Anomalopteryx_Dart: C..AAT............TA.--G.CAA.G.T------------------------------------------------------T....GT..T.A------------------

**Non-target (Soil flagellates?)**

10201_Pachyornis_Dart: C.CGA.............TC.-------------------------------------------------------AC.AA...T..T..C.TT.GAC.TG---------------

10201_Pachyornis_Dart: C.CGA.............TC.-------------------------------------------------------AC.AA...T..T..C.TT.GAC.TG---------------

10201_Pachyornis_Dart: C.CGA.............TC.-------------------------------------------------------AC.AA...T..T..C.TT.GAC.TG---------------

10201_Pachyornis_Dart: C.CGA.............TC.-------------------------------------------------------AC.AA...T..T..C.TT.GAC.TG---------------

10198_Megalapteryx_Dart: C.CGA.............TC.-------------------------------------------------------AC.AA...T..T..C.TT.GAC.TG---------------

10198_Megalapteryx_Dart: C.CGA.............TC.-------------------------------------------------------AC.AA...T..T..C.TT.GAC.TG---------------

10198_Megalapteryx_Dart: C.CGA.............TC.-------------------------------------------------------AC.AA...T..T..C.TT.GAC.TG---------------

10173_Megalapteryx_Euphrates: C.CGA.............TC.-------------------------------------------------------AC.AA...T..T..C.TT.GAC.TG---------------

10164_Megalapteryx_Euphrates: C.CGA.............TC.-------------------------------------------------------AC.AA...T..T..C.TT.GAC.TG---------------

10163_Megalapteryx_Euphrates: C.CGA.............TC.-------------------------------------------------------AC.AA...T..T..C.TT.GAC.TG---------------

10146_Megalapteryx_Euphrates: C.CGA.............TC.-------------------------------------------------------AC.AA...T..T..C.TT.GAC.TG---------------

10146_Megalapteryx_Euphrates: C.CGA.............TC.-------------------------------------------------------AC.AA...T..T..C.TT.GAC.TG---------------

10142_Megalapteryx_Euphrates: C.CGA.............TC.-------------------------------------------------------AC.AA...T..T..C.TT.GAC.TG---------------

10501_Anomalopteryx_Dart: C.CGA.............TC.--A.AA-----------------------------------------------------A...T..T.CC.TT.GTAC...--------------

10201_Pachyornis_Dart: A.CGA.............TC.--A.AA-----------------------------------------------------A...T..T..C.TT.G-AC...--------------

10142_Megalapteryx_Euphrates: C.CGA.............TC.--G.GA-----------------------------------------------------A...T.....C.T..TTC.TG---------------

10501_Anomalopteryx_Dart: C.CGA.............TC.GAT.AGA.T.C---------------------------------------------------GG..TGGT.A..A.-------------------

10501_Anomalopteryx_Dart: C..AA...AT...T....TAT----------------------------------------------------------.C.A.C.T.AGC.T..G.TCT.C...TT.GT.GGCA-

10501_Anomalopteryx_Dart: C..AA...AT...T....TAT----------------------------------------------------------.C.A.C.T.AGC.T..G.TCT.C...TT.GT.GGCA-

**Non-target (Plant nematode?)**

10197_Megalapteryx_Dart: --.AA...A........CTGA--CACA.TT.CAAG....TTCA-------------------------------------------------------------------------

10197_Megalapteryx_Dart: .C.AA...A........CTGA--CACA.TT.CAAG....TTCA-------------------------------------------------------------------------

10197_Megalapteryx_Dart: .C.AA...A........CTGA--CACA.TT.CAAG....TTCA-------------------------------------------------------------------------

10197_Megalapteryx_Dart: .C.AA...A........CTGA--CACA.TT.CAAG....TTCA-------------------------------------------------------------------------
