# Supplementary material for: A Megafauna’s Microfauna: Gastrointestinal Parasites of New Zealand’s Extinct Moa (Aves: Dinornithiformes)
Source: PLoS One. 2013 Feb 25;8(2):e57315. doi: 10.1371/journal.pone.0057315 (PMC3581471; doi:10.1371/journal.pone.0057315)
Supplement: Figure S6 — Alignment of clone sequences obtained from moa coprolites using Nem18SlongF and Nem18SlongR primers. (DOC) [file pone.0057315.s006.doc]

1 150

Consensus: CGCGGTAATTCCAGCTCCAATAGCGTATATTAAAGTTGTTGCRGTTAAAAAGCTCGTAGTTGAAMMT-GSNCYWSRCTGTTCGGTCCKYCYNAT--CGCGYGAACWGAWY---CSS-NGGGYYTGTC--------YTKTTGGGKWKCCYT

ACG-------T--CCTTRAYYGGTTGCRKRRGGKAACYAR--------CRMKTTTACTTTGAAAAAATTAGAGTGTTTAAAGCAGGC-YTWTGC-TTGAATACTYSAGCATGGAATAATAGAATAGGACYT-CGGTTCTATTTTGTTGG- TTTCYAGGWCTGARGTAATGATTAATAGGGACAGTCGGGGGCATTCGTATTGSWKYGTCAGAGGTGAAATTCTT-GGATTKWWTCAAGACGAMCTACTGCGAAAGCATTTGCCAAGRATGTYTTCAT

**Sequence 3 (Eimeriorina sp. 2)**

10503_Dinornis_Dart: ..........................................A.......C.............CT.TTGCTGTGCGAAACT..G.AGT.TT.G--GA.TT.TT.CTTGTTTCTGACA.CATTAT..--------TGA..CT.CAT.AGC

.A.----------.G..G.T--..G.TGTCA.AG----------------ATG..........T...C..........C........ATAAC..C.........CC..............AGTG..A..TC-A......-.C.......-

-...A..AG.CAGA.....................TA..........A...TGGTA.CT...............-A....TACCA.......A...............C.....G.G....T.----

**Sequence group 4 (Heterakoidea)**

10504_Megalapteryx_Dart: .................TC.A..T......CGTCA...C...G..................TG.AA.-.CG.TACAGGA........GT.CT..G-G...T....T..AC---.CC-T...CT...A--------T.G....TTTT..T.

...-------.TA....G.TC......GTAA..TG..T.A--------.GAG.......................C....T..G...-T.A...-C......T.CGT............G........TC.-.................- ....T...A.C.CT.................T..........G.C......CAATT.....G............-.....TAT.G.......A----------------------------------

10503_Dinornis_Dart: .................TC.A..T......CGTCA...C...G..C...............TG.AA.-.CG.TACAGGA........GT.CT..G-G...T....T..AC---.CC-T...CT...A--------T.G....TTTT..T.

T..-------.TA....G.TC......GTAA..TG..T.A--------.GAG.......................C....C..G...-T.A...-C......T.CGT............G........TC.-.................- ...TCT.AT....GA.....G....G........A...............C.CTGC..G...............-...CCGTAG.G....-------------------------------------

10503_Dinornis_Dart: .................TC.A..T......CGTCA...C...G..................TG.AA.-.CG.TACAGGA........GT.CT..G-G...T....T..AC---.CC-T...CT...A--------T.G....TTTT..T.

T..-------.TA....G.TC......GTAA..TG..T.A--------.GAG.......................C....C..G...-T.A...-C......T.CGT............G........TC.-.................- ...TCT.AT....GA.....G....G........A...............C.CTGC..G.............C.-...CCGTAG.G.....CC.G--------------------------------

10503_Dinornis_Dart: .................TC.A..T......CGTCA...C...G..................TG.AA.-.CG.TACAGGA........GT.CT..G-G...T....T..AC---.CC-T...CT...A--------TAG....TTTT..T.

...-------.TA....G.TC......GTAA..TG..T.A--------.GAG.......................C....T..G...-T.A...-C......T.CGT............G........TC.-.................- ...TCT.AT....GA.....G....G........A...............C.CTGC..G...............-...CCGTAG.G.....CC.G.......-------------------------

10503_Dinornis_Dart: .................TC.A..T......CGTCA...C...G..................TG.AA.-.CG.TACAGGA........GT.CT..G-G...T....T..AC---.CC-T...CT...A--------T.G....TTTT..T.

...-------.TA....G.TC......GTAA..TG..T.A--------.GAG.......................C....C..G...-T.A...-C......T.CGT............G........TC.-.................- ...TCT.AT....GA.....G....G........A...............C.CTGC..G...............-...CCGTAG.G.....CC.G.....................A....C-----

10504_Megalapteryx_Dart: .................TC.A..T......CGTCA...C...G..................TG.AA.-.CG.TACAGGA........GT.CT..G-G...T....T..AC---.CC-T...CT...A--------T.G....TTTT..T.

...-------.TA....G.TC......GTAA..TG..T.A--------.GAG.......................C....C..G...-T.A...-C......T.CGT............G........TC.-.................- ...TCT.AT....GA.....G....G........A...............C.CTGC..G...............-...CCGTAG.G.....CC.G.....................A....C-----

10503_Dinornis_Dart: .................TC.A..T......CGTCA...C...G..................TG.AA.-.CG.TACAGGA........GT.CT..G-G...T....T..AC---.CC-T...CT...A--------T.G....TTTT..T.

T..-------.TA....G.TC......GTAA..TG..T.A--------.GAG.......................C....T..G...-T.A...-C......T.CGT............G........TC.-.................- ...TCT.AT....GA.....G....G........A...............C.CTGC..G...............-...CCGTAG.G.....CC.G.....................A....C-----

10503_Dinornis_Dart: .................TC.A..T......CGTCA...C...G..................TG.AA.-.CG.TACAGGA........GT.CT..G-G...T....T..AC---.CC-T...CT...A--------T.G....TTTT..T.

T..-------.TA....G.TC......GTAA..TG..T.A--------.GAG.......................C....C..G...-T.A...-C......T.CGT............G........TC.-.................- ...TCT.AT....GA.....G....G........A...............C.CTGC..G...............C...CCGTAG.G.....------------------------------------

10503_Dinornis_Dart: .................TC.A..T......CGTCA...C...G..................TG.AA.-.CG.TACAGGA........GT.CT..G-G...T....T..AC---.CC-T...CT...A--------T.G....TTTT..T.

T..-------.TA....G.TC......GTAA..TG..T.A--------.GAG.......................C....C..G...-T.A...-C......T.CGT............G........TC.-.................- ...TCT.AT....GA.....G....G........A...............C.CTGC..G...............-...CCGTAG.G.....CC.G.....................A....C-----

10503_Dinornis_Dart: .................TC.A..T......CGTCA...C...G..................TG.AA.-.CG.TACAGGA........GT.CT..G-G...T....T..AC---.CC-T...CT...A--------T.G....TTTT..T.

T..-------.TA....G.TC......GTAA..TG..T.A--------.GAG.......................C....C..G...-T.A...-C......T.CGT............G........TC.-.................- ...TCT.AT....GA.....G....G........A...............C.CTGC..G...............-...CCGTAG.G.....CC.G.....................A....C.----

10503_Dinornis_Dart: .................TC.A..T......CGTCA...C...G..................TG.AA.-.CG.TACAGGA........GT.CT..G-G...T....T..AC---.CC-T...CT...A--------T.G....TTTT..T.

T..-------.TA....G.TC......GTAA..TG..T.A--------.GAG.......................C....C..G...-T.A...-C......T.CGT............G........TC.-.................- ...TCT.AT....GA.....G....G........A...............C.CTGC..G...............-...C------------------------------------------------

10503_Dinornis_Dart: ------------------------------------..C...G..................TG.AA.-.CG.TACAGGA........GT.CT..G-G...T....T..AC---.CC-T...CT...A--------T.G....TTTT..T.

...-------.TA....G.TC......GTAA..TG..T.A--------.GAG.......................C....T..G...-A.A...-C......T.CGT............G........TC.-.................- ...TCT.AT....GA.....G....G........A...............C.CTGC..G...............-...CCGTAG.G.....CC.G.....................A....C-----

10503_Dinornis_Dart: .................TC.A..T......CGTCA...C...G..................TG.AA.-.CG.TACAGGA........GT.CT..G-G...T....T..AC---.CC-T...CT...A--------T.G....TTTT..T.

...-------.TA....G.TC......GTAA..TG..T.A--------.GAG.......................C....T..G...-A.A...-C......T.CGT............G........TC.-.................- ...TCT.AT....GA.....G....G........A...............C.CTGC..G...............-...CCGTAG.G.....CC.G.....................A....C-----

10503_Dinornis_Dart: .................TC.A..T......CGTCA...C...G..................TG.AA.-.CG.TACAGGA........GT.CT..G-G...T....T..AC---.CC-T...CT...A--------T.G....TTTT..T.

...-------.TA....G.TC......GTAA..TG..T.A--------.GAG.......................C....T..G...-A.A...-C......T.CGT............G........TC.-.................- ...TCT.AT....GA.....G....G........A...............C.CTGC..G...............-...CCGTAG.G.....CC.G.....................A....C-----

10503_Dinornis_Dart: .................TC.A..T......CGTCA...C...G..................TG.AA.-.CG.TACAGGA........GT.CT..G-G...T....T..AC---.CC-T...CT...A--------T.G....TTTT..T.

...-------.TA....G.TC......GTAA..TG..T.A--------.GAG.......................C....T..G...-A.A...-C......T.CGT............G........TC.-.................- ...TCT.AT....GA.....G....G........A...............C.CTGC..G...............-...CCGTAG.G.....CC.G.....................A....C-----

10503_Dinornis_Dart: .................TC.A..T......CGTCA...C...G..................TG.AA.-.CG.TACAGGA........GT.CT..G-G...T....T..AC---.CC-T...CT...A--------T.G....TTTT..T.

...-------.TA....G.TC......GTAA..TG..T.A--------.GAG.......................C....T..G...-T.A...-C......T.CGT............G........TC.-.................- ...TCT.AT....GA.....G....G........A...............C.CTGC..G............A..-...CCGTAG.G.....CC.G.....................A....C-----

10504_Megalapteryx_Dart: .................TC.A..T......CGTCA...C...G..................TG.AA.-.CGTTACAGGA........GT.CT..G-G...T....T..AC---.CC-T...CT...A--------T.G....TTTT..T.

...-------.TA....G.TC......GTAA..TG..T.A--------.GAG.......................C....T..G...-T.A...-C......T.CGT............G........TC.-.................- ...TCT.AT....GA.....G....G........A...............C.CTGC..G...............-...CCGTAG.G.....CC.G.....................A....C-----

10503_Dinornis_Dart: .................TC.A..T......CGTCA...C...G..................TG.AA.-.CG.TACAGGA........GT.CT..G-G...T....T..AC---.CC-T...CT...A--------T.G....TTTT..T.

...-------.TA....G.TC......GTAA..TG..T.A--------.GAG.......................C....T..G...-T.A...-C......T.CGT............G........TC.-.................- ...TCT.AT....GA.....G....G........A...............C.CTGC..G...............-...CCGTAG.G.....CC.G............--------------------

10503_Dinornis_Dart: -------------------.A..T......CGTCA...C...G..................TG.AA.-.CG.TACAGGA........GT.CT..G-G...T....T..AC---.CC-T...CT...A--------T.G....TTTT..T.

...-------.TA....G.TC......GTAA..TG..T.A--------.GAG.......................C....T..G...-T.A...-C......T.CGT............G........TC.-.................- ...TCT.AT....GA.....G....G........A...............C.CTGC..G...............-...CCGTAG.G.....CC.G.....................A....C-----

10503_Dinornis_Dart: .................TC.A..T......CGTCA...C...G..................TG.AA.-.CG.TACAGGA........GT.CT..G-G...T....T..AC---.CC-T...CT...A--------T.G....TTTT..T.

...-------.TA....G.TC......GTAA..TG..T.A--------.GAG.......................C....T..G...-T.A...-C......T.CGT............G........TC.-.................- ...TCT.AT....GA.....G....G........A...............C.CTGC..G...............-...CCGTAG.G.....CC.G.....................A....C-----

10503_Dinornis_Dart: .................TC.A..T......CGTCA...C...G..................TG.AA.-.CG.TACAGGA........GT.CT..G-G...T....T..AC---.CC-T...CT...A--------T.G....TTTT..T.

...-------.TA....G.TC......GTAA..TG..T.A--------.GAG.......................C....T..G...-T.A...-C......T.CGT............G........TC.-.................- ...TCT.AT....GA.....G....G........A...............C.CTGC..G...............-...CCGTAG.G.....CC.G.....................A....C-----

10503_Dinornis_Dart: .................TC.A..T......CGTCA...C...G..................TG.AA.-.CG.TACAGGA........GT.CT..G-G...T....T..AC---.CC-T...CT...A--------T.G....TTTT..T.

...-------.TA....G.TC......GTAA..TG..T.A--------.GAG.......................C....T..G...-T.A...-C......T.CGT............G........TC.-.................- ...TCT.AT....GA.....G....G........A...............C.CTGC..G...............-...CCGTAG.G.....CC.G.....................A....C-----

10503_Dinornis_Dart: .................TC.A..T......CGTCA...C...G..................TG.AA.-.CG.TACAGGA........GT.CT..G-G...T....T..AC---.CC-T...CT...A--------T.G....TTTT..T.

...-------.TA....G.TC......GTAA..TG..T.A--------.GAG.......................C....T..G...-T.A...-C......T.CGT............G........TC.-.................- ...TCT.AT....GA.....G....G........A...............C.CTGC..G...............-...CCGTAG.G.....CC.G.....................A....C.....

**Non-parasite (Fungi, type 1)**

10504_Megalapteryx_Dart: ............G.............................C.....................CC.T.GGTCTGG...GC......GC.TC.C--....T.C..T.GTC---.GGCT..ATC.T..--------C.TC....GAG..CC

.T.-------G--....C.CT..CC.TGG-G..G...C.G--------GACT......G..................C.........-C.T...-.C.G....ATT.......................G.G.................- ....T...A.C.CC.................TG.........G.CA.....CGGCT.................------------------------------------------------------

10504_Megalapteryx_Dart: ..........................................A.....................AC.T.GGTCTGG...GC......GC.TC.C--....T.C..T.GTC---.GGCT..ATC.T..--------C.TC....GAG..CC

.T.-------G--....C.CT..CC.TGG-G..G...C.G--------GACT......G..................C.........-C.T...-.C.G....ATT.......................A.-.................- ....T...A.C.CT.................T..........G.C......CAATT..................-.....TAT.G.......A................C......G....T-----

10504_Megalapteryx_Dart: ..........................................A.....................CC.T.GGTCTGG...GC......GC.TC.C--....T.C..T.GTC---.GGCT..ATC.T..--------C.TC....GAG..CC

.T.-------G--....C.CT..CC.TGG-G..G...C.G--------GACT......G..................C.........-C.T...- .C.G....ATT.......................G.G................-

....T...A.C.CT.................T..........G.C......CAATT..................-.....TAT.G.......A................C......G....T-----

10504_Megalapteryx_Dart: ..........................................A.....................CC.T.GGTCTGG...GC......GC.TC.C--....T.C..T.GTC---.GGCT..ATC.T..--------C.TC....GAG..CC

.T.-------G--....C.CT..CC.TGG-G..G...C.G--------GACT......G..................C.........-C.T...-.C.G....ATT.......................G.G.................- ..C.T...A.C.CT.................T..........G.C......CAATT..................-.....TAT--------------------------------------------

**Non-parasite (Fungi, type 2)**

10504_Megalapteryx_Dart: ..........................................C.....................CC.T.GA.CTGG...A..T....TC.TA..--..A.C.T..G..TT---.GGTC...TC.T..--------C.TC....GAG..C.

.T.-------.--....T.CT...C.TAGCG..G...C.G--------GACT.....C............A......C.........-C.T...-.C......ATT.......................A.-.................- ....T...A.C.CC.................TG.........G.CA.....CGGCT...............T..-.....TGCCG.....T.A................C..---------------

10504_Megalapteryx_Dart: ..........................................C.....................CC.T.GA.CTGG...A..T....TC.TA..--..A.C.T..G..TT---.GGTC...TC.T..--------C.TC....GAG..C.

.T.-------.--....T.CT...C.TAGCG..G...C.G--------GACT.....C...................C.........-C.T...-.C......ATT.......................A.-.................- ....T...A.C.CC..............................CA.....CAATT..................-.....TAT.G.......A................C-----------------

10504_Megalapteryx_Dart: ..........................................C.....................CC.T.GA.CTGG...A..T....TC.TA..--..A.C.T..G..TT---.GGTC...TC.T..--------C.TC....GAG..C.

.T.-------.--....T.CT...C.TAGCG..G...C.G--------GACT.....C...................C.........-C.T...-.C......ATT.......................A.-.................- ....T...A.C.CC..............................CA.....CAATT..................-.....TAT.G.......A................C......G....T.----

10504_Megalapteryx_Dart: ..........................................C.....................CC.T.GA.CTGG...A..T....TC.TA..--..A.C.T..G..TT---.GGTC...TC.T..--------C.TC....GAG..C.

.T.-------.--....T.CT...C.TAGCG..G...C.G--------GACT.....C...................C.........-C.A...-.C.G....ATT.......................G.G.................- ....T...A.C.CT.................T..........G.C......CAATT..................-.....TAT.G.......A................C......G....T-----

10504_Megalapteryx_Dart: ..........................................C.....................CC.T.GA.CTGG...A..T....TC.TA..--..A.C.T..G..TT---.GGTC...TC.T..--------C.TC....GAG..C.

.T.-------.--....T.CT...C.TAGCG..G...C.G--------GACT.....C...................C.........-C.T...-.C.G....ATT.......................G.G.................- ....T...A.C.CT.................T..........G.C......CAATT..................-.....TAT.G.......A................C......G..--------

10504_Megalapteryx_Dart: ..........................................C.....................CC.T.GA.CTGG...A..T....TC.TA..--..A.C.T..G..TT---.GGTC...TC.T..--------C.TC....GAG..C.

.T.-------.--....T.CT...C.TAGCG..G...C.G--------GACT.....C...................C.........-C.T...-.C......ATT.......................A.-.................- ....T...A.C.CT.................T............C......CAATT..................-.....TAT.G.....-------------------------------------

10504_Megalapteryx_Dart: ..........................................C.....................CC.T.GA.CTGG...A..T....TC.TA..--..A.C.T..G..TT---.GGTC...TCCT..--------C.TC....GAG..C.

.T.-------.--....T.CT...C.TAGCG..G...C.G--------GACT.....C...................C.........-C.T...-.C......ATT.......................A.-.................- ....T...A.C.CT.................T..........G.C......CAATT..................-.....TAT.G.......A................C.----------------

10504_Megalapteryx_Dart: ..........................................C.....................CC.T.GA.CTGG...G..T....TC.TA..--..A.C.T..G..TT---.GGTC...TC.T..--------C.TC....GAG..C.

.T.-------.--....T.CT...C.TAGCG..G...C.G--------GACT.....C...................C.........-C.T...-.C......ATT.......................A.-.................- ....T...A.C.CT.................T..........G.C......CAATT..................-.....TAT.G.......A................C......G....T-----

10504_Megalapteryx_Dart: ..........................................C.....................CC.T.GA.CTGG...A..T....TC.TA..--..A.C.T..G..TT---.GGTC...TC.T..--------C.TC....GAG..C.

.T.-------.--....T.CT...C.TAGCG..G...C.G--------GACT.....C...................C.........-C.T...-.C......ATT.......................A.-.................- ....T...A.C.CT.................T..........G.C......CAATT..................-.....TAT.G.......A................C......G....T-----

10504_Megalapteryx_Dart: ..........................................C.....................CC.T.GA.CTGG...A..T....TC.TA..--..A.C.T..G..TT---.GGTC...TC.T..--------C.TC....GAG..C.

.T.-------.--....T.CT...C.TAGCG..G...C.G--------GACT.....C...................C.........-C.T...-.C......ATT.......................A.-.................- ....T...A.C.CT.................T..........G.C......CAATT..................-.....TAT.G.......A................C......G....T-----

10504_Megalapteryx_Dart: ..........................................C.....................CC.T.GA.CTGG...A..T....TC.TA..--..A.C.T..G..TT---.GGTC...TC.T..--------C.TC....GAG..C.

.T.-------.--....T.CT...C.TAGCG..G...C.G--------GACT.....C...................C.........-C.T...-.C......ATT.......................A.-.................- ....T...A.C.CT.................T..........G.C......CAATT..................-.....TAT.G.......A................C......G....T-----

10504_Megalapteryx_Dart: ..........................................C.....................CC.T.GA.CTGG...A..T....TC.TA..--..A.C.T..G..TT---.GGTC...TC.T..--------C.TC....GAG..C.

.T.-------.--....T.CT...C.TAGCG..G...C.G--------GACT.....C...................C.........-C.T...-.C......ATT.......................A.-.................- ....T...A.C.CT.................T..........G.C......CAATT..................-.....TAT.G.......A................C......G....T-----

10504_Megalapteryx_Dart: ..........................................C.....................CC.T.GA.CTGG...A..T....TC.TA..--..A.C.T..G..TT---.GGTC...TC.T..--------C.TC....GAG..C.

.T.-------.--....T.CT...C.TAGCG..G...C.G--------GACT.....C...................C.........-C.T...-.C......ATT.......................A.-.................- ....T...A.C.CT.................T..........G.C......CAATT..................-.....TAT.G.......A................C......G....T-----

10504_Megalapteryx_Dart: ..........................................C.....................CC.T.GA.CTGG...A..T....TC.TA..--..A.C.T..G..TT---.GGTC...TC.T..--------C.TC....GAG..C.

.T.-------.--....T.CT...C.TAGCG..G...C.G--------GACT.....C...................C.........-C.T...-.C......ATT.......................A.-.................- ....T...A.C.CT.................T..........G.C......CAATT..................-.....TAT.G.......A................C......G....T-----

10504_Megalapteryx_Dart: ..........................................C.....................CC.T.GA.CTGG...A..T....TC.TA..--..A.C.T..G..TT---.GGTC...TC.T..--------C.TC....GAG..C.

.T.-------.--....T.CT...C.TAGCG..G...C.G--------GACT.....C...................C.........-C.T...-.C......ATT.......................A.-.................- ....T...A.C.CT.................T..........G.C......CAATT..................-.....TAT.G.......A.............---------------------

**Sequence 2 (Eimeriorina)**

10504_Megalapteryx_Dart: ................................G.........A...................G.TT.---------.....G..ATTTT.AGG.AC...CT.T.AT.GGTGTG.GCTTC.ATT.A..TCGGCATTT.TCC..TGAG..A.

T..CGCTTAA.TG.G..GTTT....TTTTCC..A.---------------CT..........G....A..........C........TT.TC..T.........GC..............AG.......T.-T................- ....T...A..A.A.....................T...............TAACT..................-A....T----------------------------------------------

**Sequence 1 (*Cryptosporidium*)**

10198_Megalapteryx_Dart: ..........................................A.....................TT.---------.........T.T--------...TC...GAC.TT-------------....--------AAC...C.CGA..GA

..A-------.--.A.GA.C---....ACGGA.C..TCCGCGTATGTT.ACC..........G...............C.....A..-G.CC..T.........AC..............C........T.-TA.GC....-.......-

....-...C....A.....................T................GATA.....C........G.G.-..--------------------------------------------------

10198_Megalapteryx_Dart: ..........................................A.....................TT.---------.........T.T--------...TC...GAC.TT-------------....--------AAC...C.CGA..GA

..A-------.--.A.GA.C---....ACGT..C...ACGCGTATGTT.ACC..........G...............C.....A..-G.CC..T.........AC..............C........T.-...GC....-.......-

....-T..C....A.....................T...............CGACA.....C........G...-T....TGT.G.....-------------------------------------

10198_Megalapteryx_Dart: ..........................................A.......G.............TT.---------.........T.T--------...TC...GAC.TT-------------....--------AAC...C.CGA..GG

..A-------.--.A.GA.C---....ACGT..T...ACGCGTATGTT.ACC..........G...............C.....A..-G.CC..T.........AC..............C........T.-TA.GC....-.......-

....-...C....A.....................T................GATA.....C........G.G.-...-------------------------------------------------

10198_Megalapteryx_Dart: ..........................................A.....................TT.---------.........T.T--------...TC...GAC.TT-------------....--------AAC...C.CGA..GA

..A-------.--.A.GA.C---....ACGT..C...ACGCGTATGTT.ACC..........G...............C.....A..-G.CC..T.........AC..............C........T.-TA.GC....-.......- ....T...A..A.A.....................T................GATA.....C........G.G.-.....TAT.........A......................AG----------

10198_Megalapteryx_Dart: ..........................................A.....................TT.---------.........T.T--------...TC...GAC.TT-------------....--------AAC...C.CGA..GA

..A-------.--.A.GA.C---....ACGT..C...ACGCGTATGTT.ACC..........G...............C.....A..-G.CC..T.........AC..............C........T.-TA.GC....-.......- ....T...A..A.A.....................T................GATA.....C........G.G.-.....TAT.........A.......---------------------------

10198_Megalapteryx_Dart: .....................G....................A.......G.............TT.---------.........T.T--------...TC...GAC.TT-------------....--------AAC...C.CGA..GA

..A-------.--.A.GA.C---....ACGT..C...ACGCGTATGTT.ACC..........G...............C.....A..-G.CC..T.........AC..............C........T.-TA.GC....-.......-

....-...C....A.....................T................GATA.....C........G.G.-.....TAT.........A......................AG....T-----

10198_Megalapteryx_Dart: ..........................................A.....................TT.---------.........T.T--------...TC...GAC.TT-------------....--------AAC...C.CGA..GA

..A-------.--.A.GA.C---....ACGT..C...ACGCGTATGTT.ACC..........G...............C.....A..-G.CC..T.........AC..............C........T.-TA.GC....-.......-

....-...C....A.....................T.......G........GATA.....C........G...-T....TGT.G.......A......................AG....T-----

10198_Megalapteryx_Dart: ..........................................A.....................TT.---------.........T.T--------...TC...GAC.TT-------------....--------AAC...C.CGA..GA

..A-------.--.A.GA.C---....ACGT..C...ACGCGTATGTT.ACC..........G...............C.....A..-G.CC..T.........AC..............C........T.-TA.GC....-.......-

....-...C....A..................G..T................GATA.....C........G.G.-.....TAT.........A......................AG....T.----

10198_Megalapteryx_Dart: ..........................................A.....................TT.---------.........T.T--------...TC...GAC.TT-------------....--------AAC...C.CGA..GA

..A-------.--.A.GA.C---....ACGT..C...ACGCGTATGTT.ACC..........G...............C.....A..-G.CC..T.........AC..............C........T.-TA.GC....-.......-

....-...C....A.....................T................GATA.....C.A......G.G.-.....TAT...-----------------------------------------

10198_Megalapteryx_Dart: ..........................................A.....................TT.---------.........T.T--------...TC...GAC.TT-------------....--------AAC...C.CGA..GA

..A-------.--.A.GA.C---....ACGT..C...ACGCGTATGTT.ACC..........G...............C.....A..-G.CC..T.........AC..............C........T.-TA.GC....-.......-

....-...C....A.....................T..............-----------------------------------------------------------------------------

10198_Megalapteryx_Dart: ..........................................A.....................TT.---------.........T.T--------...TC...GAC.TT-------------....--------AAC...C.CGA..GA

..A-------.--.A.GA.C---....ACGT..C...ACGCGTATGTT.ACC..........G...............C.....A..-G.CC..T.........AC..............C........T.-TA.GC....-.......-

....-...C....A.....................T................GATA.....C........G.G.-.....TAT.........A......................AG....T-----

10198_Megalapteryx_Dart: ..........................................A.....................TT.---------.........T.T--------...TC...GAC.TT-------------....--------AAC...C.CGA..GA

..A-------.--.A.GA.C---....ACGT..C...ACGCGTATGTT.ACC..........G...............C.....A..-G.CC..T.........AC..............C........T.-TA.GC....-.......-

....-...C....A.....................T................GATA.....C........G.G.-.....TAT.........A......................AG....T-----

10198_Megalapteryx_Dart: ..........................................A.....................TT.---------.........T.T--------...TC...GAC.TT-------------....--------AAC...C.CGA..GA

..A-------.--.A.GA.C---....ACGT..C...ACGCGTATGTT.ACC..........G...............C.....A..-G.CC..T.........AC..............C........T.-TA.GC....-.......-

....-...C....A.....................T................GATA.....C........G.G.-.....TAT.........A......................AG....T-----

10198_Megalapteryx_Dart: ..........................................A.....................TT.---------.........T.T--------...TC...GAC.TT-------------....--------AAC...C.CGA..GA

..A-------.--.A.GA.C---....ACGT..C...ACGCGTATGTT.ACC..........G...............C.....A..-G.CC..T.........AC..............C........T.-TA.GC....-.......-

....-...C....A.....................T................GATA.....C........G.G.-.....TAT.........A......................AG----------

10198_Megalapteryx_Dart: ..........................................A.....................TT.---------.........T.T--------...TC...GAC.TT-------------....--------AAC...C.CGA..GA

..A-------.--.A.GA.C---....ACGT..C...ACGCGTATGTT.ACC..........G...............C.....A..-G.CC..T.........AC..............C........T.-TA.GC....-.......-

....-...C....A.....................T................GATA.....C......-----------------------------------------------------------

10198_Megalapteryx_Dart: ..........................................A.....................TT.---------.........T.T--------...TC...GAC.TT-------------....--------AAC...C.CGA..GA

..A-------.--.A.GA.C---....ACGT..C...ACGCGTATGTT.ACC..........G...............C.....A..-G.CC..T.........AC..............C........T.-TA.GC....-.......-

....-...C....A.....................T................GATA.....C........G.G.-.....TAT.........A......................AG..--------

10198_Megalapteryx_Dart: ..........................................A.....................TT.---------.........T.T--------...TC...GAC.TT-------------....--------AAC...C.CGA..GA

..A-------.--.A.GA.C---....ACGT..C...ACGCGTATGTT.ACC..........G...............C.....A..-G.CC..T.........AC..............C........T.-TA.GC....-.......-

....-...C....A.....................T................GATA.....C........G.G.-.....TAT.........A....................--------------
