# Supplementary figures and images for: A Megafauna’s Microfauna: Gastrointestinal Parasites of New Zealand’s Extinct Moa (Aves: Dinornithiformes)
Source: PLoS One. 2013 Feb 25;8(2):e57315. doi: 10.1371/journal.pone.0057315 (PMC3581471; doi:10.1371/journal.pone.0057315)

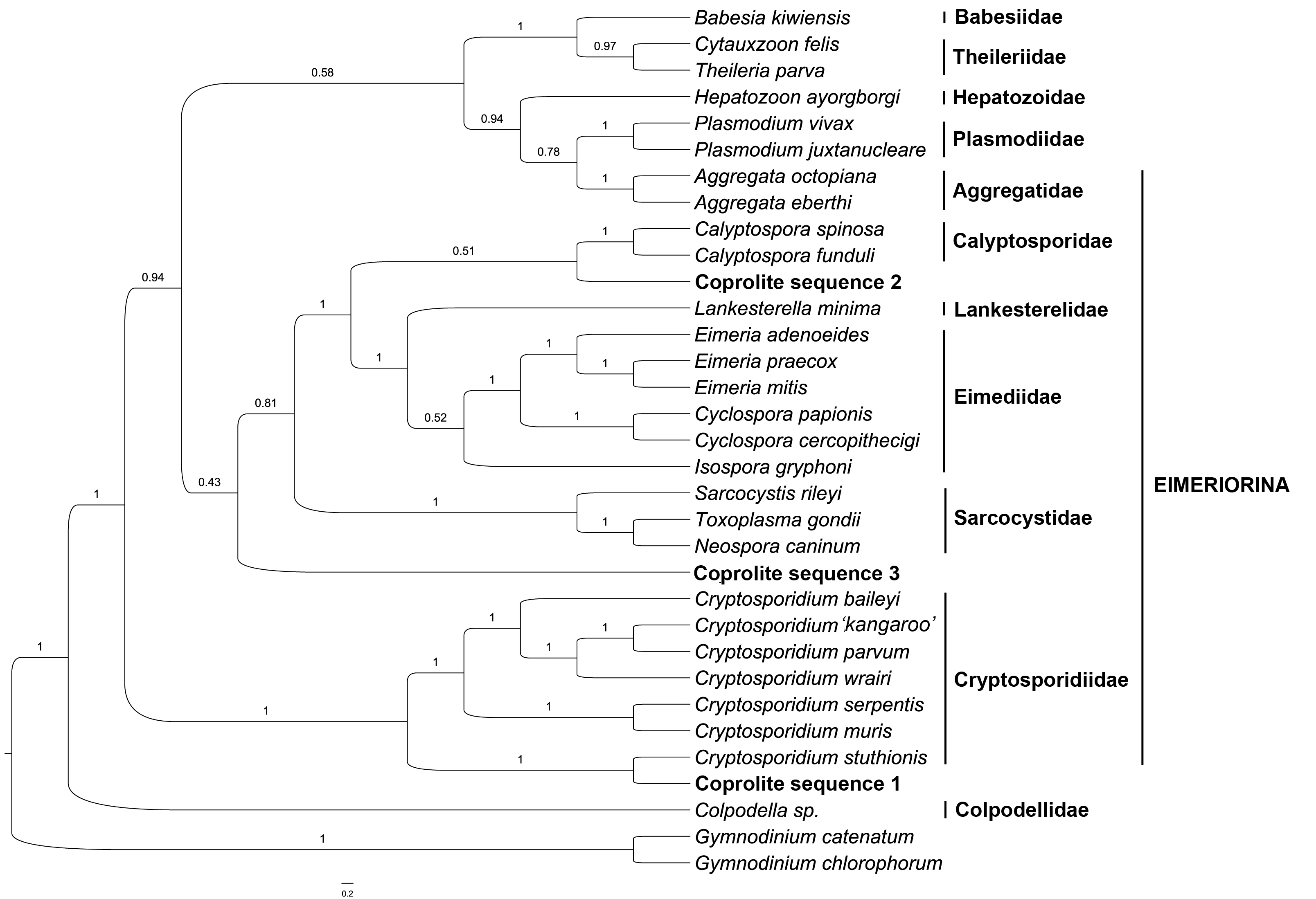

Supplement: Figure S7 — Maximum-credibility tree for 18S sequences of representative Apicomplexa (from Genbank), and moa coprolite sequences 1–3. The tree is rooted with Gymnodium (Dinoflagellata). (JPG) [file pone.0057315.s007.jpg]

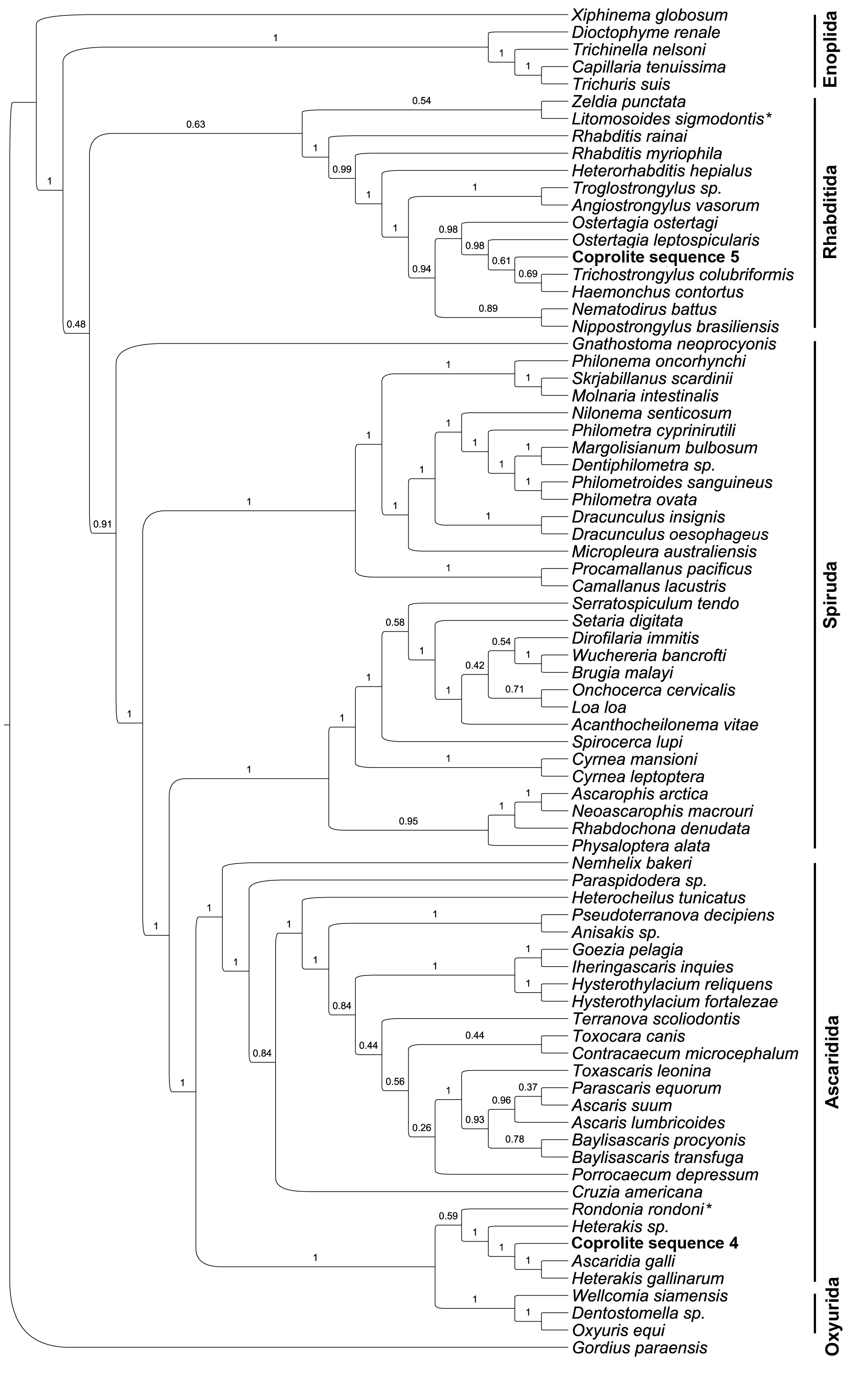

Supplement: Figure S8 — Maximum-credibility tree for 18S sequences of representative Nematoda (from Genbank), and moa coprolite sequences 4–5. The tree is rooted with Gordius (Nematomorpha). All sequences are correctly resolved within clades representing 5 major orders of parasitic nematodes, except *Litomosoides (Spiruda) and Rondonia (Rhabditida). (JPG) [file pone.0057315.s008.jpg]

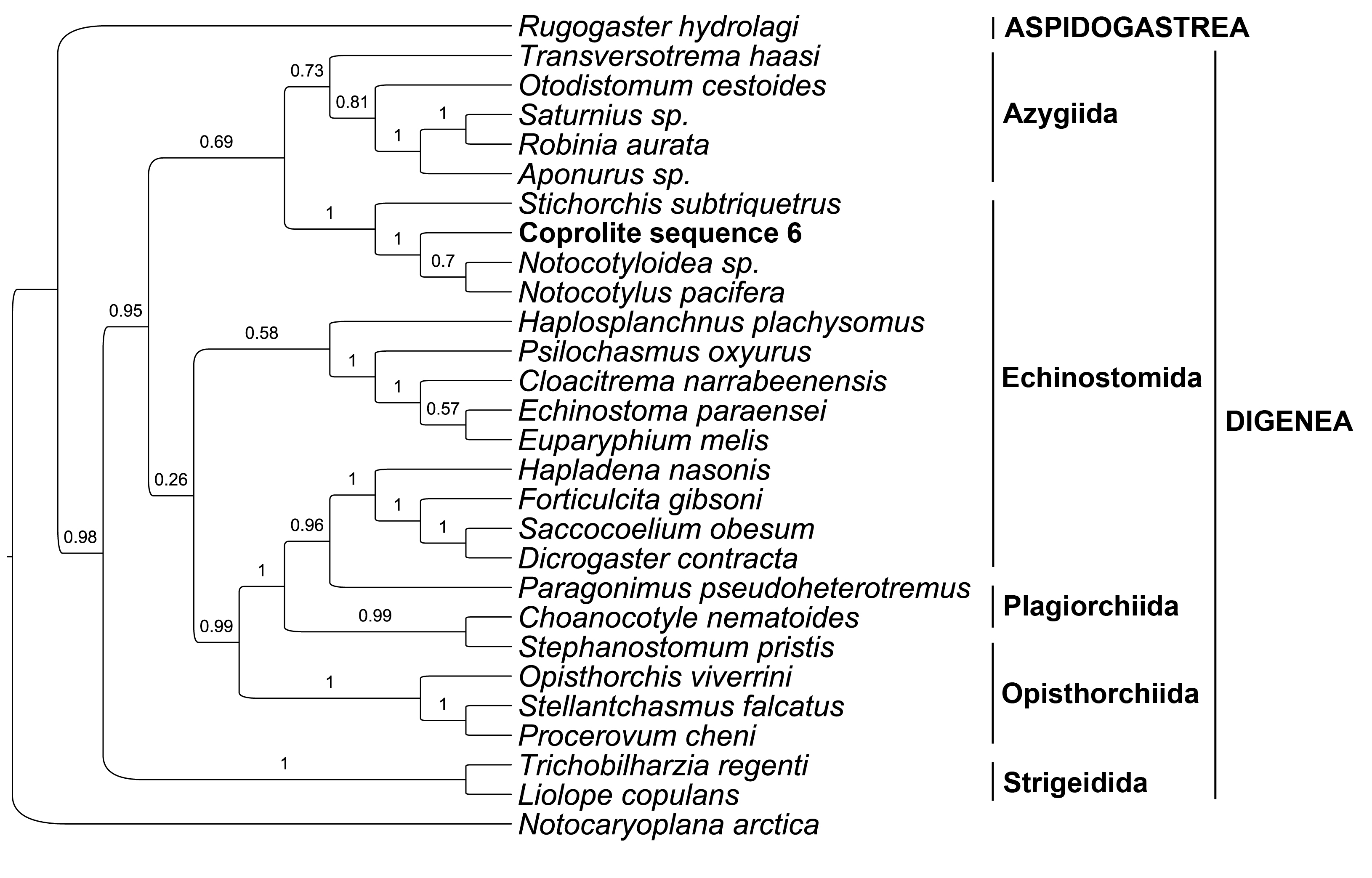

Supplement: Figure S9 — Maximum-credibility tree for 18S sequences of representative Trematoda (from Genbank), and moa coprolite sequence 6. The tree is rooted with Notocaryoplana (Turbullaria). (JPG) [file pone.0057315.s009.jpg]
