# Supplementary material for: A Megafauna’s Microfauna: Gastrointestinal Parasites of New Zealand’s Extinct Moa (Aves: Dinornithiformes)
Source: PLoS One. 2013 Feb 25;8(2):e57315. doi: 10.1371/journal.pone.0057315 (PMC3581471; doi:10.1371/journal.pone.0057315)
Supplement: Table S1 — Moa coprolite specimens that were used in this study. Specimen numbers relate to the Australian Centre for Ancient DNA sample database (A). Where voucher specimens exist, the museum registration numbers are also given (CM, Canterbury Museum, New Zealand; OM, Otago Museum, New Zealand). (DOC) [file pone.0057315.s010.doc]

**Little bush moa (*Anomalopteryx didiformis*)**

**Dart River Valley**

A10191, A10501 (CM Av43102), A10524 (CM Av43113)

**South Island giant moa (*Dinornis robustus*)**

**Dart River Valley**

A2062 (OM Av10720), A2063 (OM Av10667), A2064 (OM Av10670), A2065 (OM Av10716), A2066 (OM Av10707), A2070 (OM Av10715), A2072 (OM Av10674), A2102 (OM Av10686), A2103 (OM Av10732), A2109 (OM Av10675), A2110 (OM Av10717), A10189 (CM Av43090), A10190 (CM Av43091), A10199 (CM Av43097), A10203 (CM Av43100), A10503 (CM Av43104), A10530 (CM Av43116)

**Upland moa (*Megalapteryx didinus*)**

**Dart River Valley**

A2082 (OM Av10681), A2105 (OM Av10677), A2106 (OM Av10719), A2107 (OM Av10718), A10193 (CM Av43092), A10194 (CM Av43093), A10196 (CM Av43094), A10197 (CM Av43095), A10198 (CM Av43096), A10500 (CM Av43101), A10502 (CM Av43103), A10504 (CM Av43105), A10506 (CM Av43107), A10507 (CM Av43108), A10521 (CM Av43110), A10522 (CM Av43111), A10523 (CM Av43112), A10525 (CM Av43114), A10526 (CM Av43115)

**Euphrates Cave**

A10142 (CM Av43059), A10143 (CM Av43060), A10144 (CM Av43061), A10145 (CM Av43062), A10146 (CM Av43063), A10147 (CM Av43064), A10148 (CM Av43065), A10149 (CM Av43066), A10150 (CM Av43067), A10151 (CM Av43068), A10152 (CM Av43069), A10153 (CM Av43070), A10154 (CM Av43071), A10155 (CM Av43072), A10156, A10157 (CM Av43073), A10158, A10159 (CM Av43074), A10160 (CM Av43075), A10161 (CM Av43076), A10162 (CM Av43077), A10163 (CM Av43078), A10164 (CM Av43079), A10165 (CM Av43080), A10166 (CM Av43081), A10167 (CM Av43082), A10168 (CM Av43083), A10169 (CM Av43084), A10170 (CM Av43085), A10171, A10172 (CM Av43086), A10173, A10174 (CM Av43087), A10175 (CM Av43088), A10176 (CM Av43089)

**Heavy-footed moa (*Pachyornis elephantopus*)**

**Dart River Valley**

A2071 (OM Av10709), A2101 (OM Av10690), A2108 (OM Av10711), A10192, A10200 (CM Av43098), A10201 (CM Av43099), A10505 (CM Av43106), A10508 (CM Av43109)

**Kawarau Gorge**

A2074 (OM Av10740)

**Roxburgh Gorge**

A2069 (OM Av10750)
