# Supplementary material for: A Megafauna’s Microfauna: Gastrointestinal Parasites of New Zealand’s Extinct Moa (Aves: Dinornithiformes)
Source: PLoS One. 2013 Feb 25;8(2):e57315. doi: 10.1371/journal.pone.0057315 (PMC3581471; doi:10.1371/journal.pone.0057315)
Supplement: Table S2 — 18S sequences from GenBank that were used in the phylogenetic analyses. (DOC) [file pone.0057315.s011.doc]

**Apicomplexa**

*Aggregata eberthi* DQ096838; *A. octopiana* DQ096837; *Babesia kiwiensis* EF551335; *Calyptospora funduli* FJ904646; *C. spinosa* FJ904636; *Colpodella* sp*.* GU067926; *Cryptosporidium baileyi* DQ898161; *C. muris* L19069; *C. parvum* AF115377; *C. serpentis* AF151376; *C. wrairi* AF115378; *C.* sp. 'struthionis' AJ697751; *C*. sp. 'kangaroo' AF513227; *Cyclospora cercopithecigi* AF111184; *C. papionis* AF111187; *Cytauxzoon felis* L19080; *Eimeria adenoeides* FR745914; *E. mitis* FJ236379; *E. praecox* GQ421692; *Gymnodinium catenatum* GU362426; *G. chlorophorum* AM184122; *Hepatozoon ayorgborgi* EF157822; *Isospora gryphoni* AF080613; *Lankesterella minima* AF080611; *Neospora caninum* AJ271354; *Plasmodium juxtanucleare* AF463507; *P. vivax* PVU93233; *Sarcocystis rileyi* HM185742; *Theileria parva* L02366; *Toxoplasma gondii* L37415

**Nematoda**

*Acanthocheilonema viteae* DQ094171; *Angiostrongylus vasorum* EF514916; *Anisakis* sp. U94365; *Ascaridia galli* EF180058; *Ascaris lumbricoides* U94366; *A. suum* U94367; *Ascarophis arctica* DQ094172; *Baylisascaris procyonis* U94368; *B. transfuga* U94369; *Brugia malayi* AF036588; *Camallanus lacustris* DQ442663; *Capillaria tenuissima* EU004822*; Contracaecum microcephalum* AY702702; *Cruzia americana* U94371; *Cyrnea leptoptera* EU004815; *C. mansioni* AY702701; *Dentiphilometra* sp. DQ442673; *Dentostomella* sp. AF036590; *Dioctophyme renale* AB595139; *Dirofilaria immitis* AF036638; *Dracunculus insignis* AY947719; *D. oesophageus* AY852269; *Gnathostoma neoprocyonis* Z96947; *Goezia pelagia* U94372; *Gordius paranensis* AF421766; *Haemonchus contortus* EU086375; *Heterakis gallinarum* DQ503462; *Heterakis* sp. AF083003; *Heterocheilus tunicatus* U94373; *Heterorhabditis hepialus* AF083004; *Hysterothylacium fortalezae* U94374; *H. reliquens* U94376; *Iheringascaris inquies* U94377; *Litomosoides sigmodontis* AF227233; *Loa loa* DQ094173; *Margolisianum bulbosum* AB185161; *Micropleura australiensis* DQ442678; *Molnaria intestinalis* DQ442668; *Nematodirus battus* U01230; *Nemhelix bakeri* DQ118537; *Neoascarophis macrouri* DQ442660; *Nilonema senticosum* DQ442671; *Nippostrongylus brasiliensis* AJ920356; *Onchocerca cervicalis* DQ094174; *Ostertagia leptospicularis* AJ920351; *O. ostertagi* AJ920352; *Oxyuris equi* EF180062; *Parascaris equorum* U94378; *Paraspidodera* sp. AF083005; *Philometra cyprinirutili* DQ442675; *P. ovata* DQ442677; *Philometroides sanguineus* DQ442676; *Philonema oncorhynchi* DQ442670; *Physaloptera alata* AY702703; *Porrocaecum depressum* U94379; *Procamallanus pacificus* DQ442665; *Pseudoterranova decipiens* U94380; *Rhabditis myriophila* U13936; *R. rainai* AF083008; *Rhabdochona denudata* DQ442659; *Rondonia rondoni* DQ442679; *Serratospiculum tendo* AY702704; *Setaria digitata* DQ094175; *Skrjabillanus scardinii* DQ442669; *Spirocerca lupi* AY751497; *Terranova scoliodontis* DQ442661; *Toxascaris leonina* U94383; *Toxocara canis* U94382; *Trichinella nelsoni* AY851261; *Trichostrongylus colubriformis* AJ920350; *Troglostrongylus* sp. GU946677; *Trichuris suis* EU790668; *Wellcomia siamensis* EF180079; *Wuchereria bancrofti* AF227234; *Xiphinema globosum* GU549476; *Zeldia punctata* U61760

**Trematoda**

*Aponurus* sp. DQ354372; *Choanocotyle nematoides* EU196359; *Cloacitrema narrabeenensis* AY222134; *Dicrogaster contracta* FJ211256; *Echinostoma paraensei* FJ380226; *Euparyphium melis* AY222131; *Forticulcita gibsoni* FJ211226; *Hapladena nasonis* AY222146; *Haplosplanchnus plachysomus* FJ211224; *Liolope copulans* AB551567; *Notocaryoplana arctica* HM026568; Notocotyloidea sp. EU371601; *Notocotylus pacifera* AY245765; *Opisthorchis viverrini* HM004211; *Otodistomum cestoides* AJ287553; *Paragonimus pseudoheterotremus* HM004210; *Procerovum cheni* HM004212; *Psilochasmus oxyurus* AY222135; *Robinia aurata* DQ354371; *Rugogaster hydrolagi* AJ287573; *Saccocoelium obesum* FJ211254; *Saturnius* sp. DQ354370; *Stellantchasmus falcatus* HM004209; *Stephanostomum pristis* DQ248209; *Stichorchis subtriquetrus* AY245769; *Transversotrema haasi* AJ287583; *Trichobilharzia regenti* AY157
