# Supplementary material for: A Megafauna’s Microfauna: Gastrointestinal Parasites of New Zealand’s Extinct Moa (Aves: Dinornithiformes)
Source: PLoS One. 2013 Feb 25;8(2):e57315. doi: 10.1371/journal.pone.0057315 (PMC3581471; doi:10.1371/journal.pone.0057315)
Supplement: Table S3 — Six distinct parasite sequences obtained from moa coprolites that were used in the phylogenetic analyses. Note that the analyses of sequences 1, 2 and 4 used the concatenated sequences from both primer sets (see methods section for explanation). (DOC) [file pone.0057315.s012.doc]

**Sequence 1**: **Type coprolite, A10198 (*Megalapteryx didinus,* Dart River Valley)**

***Nem18Slong primers***

CGCGGTAATTCCAGCTCCAATAGCGTATATTAAAGTTGTTGCAGTTAAAAGGCTCGTAGTTGAATTTCTGTTCGGTTCTCGCTCGAAGACATTTGTCAACTTGCGCGACCGGACATCATGAACTTGCACGTGGTAACACGCGTATGTTCACCTTTACTTTGAGAAAATTAGAGTGTTTCAAGCAAGCGTCCGCTTTGAATACTACAGCATGGAATAATACAATAGGACTTTAGGCCTATTTGTTGGTTTCAGGCCTGAAGTAATGATTAATAGGGACAGTTGGGGGCATTCGTATTGGATAGTCAGCGGTGAAATGCGTGGATTTATTCAAGACGAACTACTGCGAAAGCATTTGCCAAAGATGTT

***Nem18S primers***

CTTAACCTGCTAAATAGTCACAGGAAATTCGTTTTCTGGTAG

**Sequence 2**: **Type coprolite, A10504 (*Megalapteryx didinus,* Dart River Valley)**

***Nem18Slong primers***

CGCGGTAATTCCAGCTCCAATAGCGTATATTAGAGTTGTTGCAGTTAAAAAGCTCGTAGTTGGATTTCTGTTGGGATTTTCAGGTACCGCCTGTAATGGGTGTGCGCTTCGATTTATCTCGGCATTTTTCCGGTGAGCCATTCGCGCTTAATTGCGTTGTTTGGTTTTTTCCGGAACTTTTACTTTGAGAAAAATAGAGTGTTTCAAGCAGGCTTTTCGCTTTGAATACTGCAGCATGGAATAATAAGATAGGACTTTGGTTCTATTTTGTTGGTTTCTAGGACTAAAGTAATGATTAATAGGGACAGTTGGGGGCATTCGTATTTAACTGTCAGAGGTGAAATTCTTAGATTT

***Nem18S primers***

CTTAGCCTGCTAAATAGGATCGGGAACTTTATGTTTCTGCATC

**Sequence 3**: **Type coprolite, A10503 (*Dinornis robustus,* Dart River Valley)**

***Nem18Slong primers***

CGCGGTAATTCCAGCTCCAATAGCGTATATTAAAGTTGTTGCAGTTAAAACGCTCGTAGTTGAACTTTTGCTGTGCGAAACTGGGCAGTCTTAGGACTTGTTCCTTGTTTCTGACAGCATTATTCTGATTCTGCATCAGCAAGCGTTGATGTGGTGTCAGAGATGTTACTTTGAATAAACTAGAGTGTTTCAAGCAGGCATAACGCCTTGAATACTCCAGCATGGAATAATAAGTGAGAACTCAGGTTCTTCTTGTTGGTTCAAGAGCCAGAGTAATGATTAATAGGGACAGTTAGGGGCATTCGAATTTGGTAGCTAGAGGTGAAATTCTTAGATTTACCAAAGACGAACTACTGCGAAAGCATCTGCCAGGGATGTTT

**Sequence 4**: **Type coprolite, A10503 (*Dinornis robustus,* Dart River Valley)**

***Nem18Slong primers***

CGCGGTAATTCCAGCTCTCAAAGTGTATATCGTCATTGCTGCGGTTAAAAAGCTCGTAGTTTGAAATGCGCTACAGGATTCGGTCCGTCCTATGGGCGTGAACTGAACCCCTGGGCTTGTATTGTTGGTTTTCCTTTCGTTACCTTGATCGGTTGCGTAAGGTGACTAACGAGTTTACTTTGAAAAAATTAGAGTGCTTAACGCGGGCTTATGCCTGAATATTCGTGCATGGAATAATGGAATAGGATCTCGGTTCTATTTTGTTGGTTTTCTGATCTGAGATAATGGTTAAGAGGGACAGACGGGGGCATTCGTATCGCTGCGTGAGAGGTGAAATTCCTGGACCGTAGCGAGACGCCCGACTGCGAAAGCATTTGCCAAGAATGTC

***Nem18S primers***

TCTGACCTACTAAATAGTGTCTGAATAATTATGTGCAGACG

**Sequence 5**: **Type coprolite, A10142 (*Megalapteryx didinus,* Euphrates Cave)**

***Nem18S primers***

TCTAGCCTGCTAAATAGTGGCTGGATTTTTAAGTCCAGTCT

**Sequence 6: Type coprolite: A10198 (*Megalapteryx didinus,* Dart River Valley)**

***Nem18S primers***

TTTTGCCTGCTAAATAGTATGCCTGTCCTCTGTGCTCGTTCAGGTCACGATATAAGCTGCCTCCTTGTGGGGTAGCGGAGTCGTTGTCCGACGGGTGCGGCGCAGGTAATT
